# Investigation of Brain Performance in the Schizophrenia Spectrum Using EEG with Mental and Temporal Effort

**DOI:** 10.3390/s26216952

**Published:** 2026-11-03

**Authors:** Bernadete Márcia Voichcoski Franco da Rosa, Pedro Kowacs, Geysa Peters, Ricardo Sbalqueiro, Rosângela Carmo, Wesley Hummig, Percy Nohama

**Affiliations:** 1 Graduate Program in Electrical Engineering and Industrial Informatics, Universidade Tecnológica Federal do Paraná (UTFPR), Curitiba 80230-901, Brazil; bernadetemarciarosa@alunos.utfpr.edu.br; 2 Instituto de Neurologia de Curitiba, INC, Curitiba 81210-310, Brazil; pkowacs@gmail.com (P.K.); geysa_peters@yahoo.com.br (G.P.); 3 Hospital San Julian, Piraquara 83301-010, Brazil; ricardosbalqueiro@yahoo.com.br (R.S.); rosangelaribeirozanza@gmail.com (R.C.); 4 Clínica Neuropsiquiátrica Avançada Wesley Hummig, Curitiba 80540-180, Brazil; contato@clinicawesleyhummig.com.br; 5 Graduate Program in Health Technology, Pontifícia Universidade Católica do Paraná (PUCPR), Curitiba 80215-901, Brazil

**Keywords:** brain potentials, EEG, mental effort, schizophrenia biopotentials, schizophrenia job, neuropsychiatry, schizophrenia cognitive

## Abstract

**Highlights:**

**What are the main findings?**

- A brain with schizophrenia can show voltage potential levels close to those of a healthy brain when properly treated.
- A brain with schizophrenia cannot maintain the same level of mental effort as a healthy brain even when treated.

**What are the implications of the main findings?**

- When the brain’s electrical potential is lower than the patterns, it can be corrected by medications.
- It is possible to accomplish the levels of the brain electrical potential by comparing medications.

**Abstract:**

Schizophrenia Spectrum Disorder, widely known as causing behavioral changes, deprives many professionals—who remain capable of working—their jobs. This study aimed to investigate the use of electroencephalography exam (EEG) combined with standardized mental effort sequence as a complementary tool for clinical diagnosis and to monitor the treatment response in individuals with schizophrenia and those who want to return to professional activities. EEG recordings were acquired before (IR_Initial Register) and after (FR_Final Register) the completion of a set of timed mental effort tasks in a group of 25 clinically stable individuals diagnosed with schizophrenia with positive symptoms and an 11-member healthy volunteer control group. Data from both groups were compared using temporal and spectral descriptors, and the results were analyzed statistically. The results revealed that, in the FR phase, the Vtotal parameter for the schizophrenia group was 7.89% lower, and the frequency parameter was 16.35% lower than in the healthy group. Notable differences were observed in the tolerance parameter for the duration of sustained cognitive effort: 57.9% of healthy volunteers sustained 100% of the effort duration, compared with 6.1% of volunteers with schizophrenia.

## 1. Introduction

Schizophrenia is a highly debilitating disease that removes many professionals from the job market every year, leading to early retirements; it is also responsible for many dropouts from schools and universities, resulting in a considerable loss of intellectual capital. Statistics indicate that 1% of the world’s population suffers from the disease, meaning that 1% of families worldwide have a compromised quality of life because of it. Schizophrenia has been the subject of scientific inquiry for over a century and continues to pose significant challenges to mental health professionals [1]. It affects both men (aged 18 to 25) and women (aged 25 to 35) but tends to be more severe in males. Frequently misdiagnosed or initially treated as depression, its etiology is multifactorial, involving a complex interplay of genetic, nutritional, metabolic, physiological, psychological, psychiatric, and environmental components [2,3].

Synaptic loss in schizophrenia is widespread and regionally variable, resulting in heterogeneous cognitive and behavioral impairment. Some individuals exhibit greater deficits in affective processing, while others exhibit deficits in mathematical reasoning or language-related functions. These impairments tend to progress over time, driven by ongoing synaptic degradation, which alters social behavior and affectivity [1]. However, well-formed brains during gestation allow them to have a longer lifespan than those that had malformations due to many factors, such as, for example, the pregnant mother’s exposure to heavy metals, pesticides, nutritional deficiencies and metabolic problems, alcohol intake and/or drug use, teratogenic drugs, and/or psychotropic drugs [4,5,6]; exposure to long periods of psychological stress [7,8], viral infections such as *Toxoplasma gondii* [9], bacterial infections such as *Bartonella henselae* [10], inflammation, and allergenic components, among others.

Schizophrenia is classified into two types, schizophrenia with positive symptoms (with a better prognosis) and schizophrenia with negative symptoms (with the worst prognosis), and many specific classifications within each type [11]. The evolution of schizophrenia with positive symptoms is better with appropriate treatment (medication, therapies, nutrition, and a healthy lifestyle) [12,13]; so, it is possible to slow deterioration, allowing people to preserve their intellectual capital and lead a productive life for longer [13,14]. In this context, many people (with schizophrenia with positive symptoms) wish to return to work after overcoming a crisis and when they feel well [15]. The challenge lies in synaptic gain and neurogenesis in schizophrenia—processes that are reduced due to frontal lobe impairment caused by the disease [14,16]—but occur slowly, as explained by Costa et al. [17]. In this context, measures such as a diet that does not induce dysbiosis; good sleep quality; stress management; and the reduction of inflammatory factors [18] are crucial for preventing mood destabilization, which can trigger acute psychotic episodes and accelerate neuronal degradation and a new crisis [1].

Alongside this, other measures—such as reducing working hours or changing job roles—can make a difference when returning to work [19,20]. However, it is important that these measures be based on mathematical scales that allow for the assessment of the individual’s condition (tracking whether there has been improvement or deterioration compared to a previous period) and help avoid errors or work overload [21,22].

One way to compare brain conditions is to subject the brain to activities that test its functionality. The brain is composed of lobes with specific functions, featuring interconnections among them and with various other areas to ensure the optimal functioning of a healthy organ and to coordinate all body actions and movements. However, as reported by Heinrichs and Zakzanis [20], schizophrenia affects various areas of brain processing, resulting in varying degrees of impairment across many cognitive domains—including the ability to sustain attention; the speed of perception and visual/auditory processing; working memory; verbal and visual learning; abstract, spatial, and numerical reasoning; pattern recognition; problem-solving; social interaction rules; and affectivity, among others, as described by Nuechterlein et al. [21]. Countless tasks can be used to test the brain, but there are validated tests in the field of psychology that serve this purpose [22]. However, for good performance, it is important that the brain be free of anemia, which can cause dizziness and malaise, as well as weakness (impairing work and concentration); it is a fact that a person with anemia experiences impaired cognitive function [23]. In brain comparison, one of the most important factors is electrical levels. Brain activity produces electrophysiological signals that are produced by a big set of neurons, whose potential can be measured on the scalp. Verifying the electrophysiological conditions of the brain is fundamental to evaluating whether it is healthy and whether it will sustain the effort of the reasoning that real life or work requires [24,25]. These reasoning processes are simple in healthy brains but not so simple for brains that have some disease, like schizophrenia. In this scenario, electroencephalography (EEG) is used to assess brain activity, which has the advantage that it does not use contrast agents that are toxic to the kidneys and liver [26] and so can be repeated more frequently to assess medication effects and verify other treatment evolutions. EEG measures brain electrical activity in real time in terms of amplitude and frequency, offering information that will can be EEG biomarkers for schizophrenia. A brain with schizophrenia can exhibit electrical activity like that of a healthy brain, but subtle differences have been observed in comparison with healthy brains [27,28,29].

Significant differences have been observed in patients with schizophrenia compared with healthy controls [30,31,32] in delta (0.5–4 Hz), theta (4.125–8 Hz), and lower alpha (8.125–13 Hz) activity [33,34,35]. In delta waves (sleep or anesthesia), studies have reported increased power [33]. In theta waves, an increase of power has mainly been observed in the frontal lobe [34]. Some important differences have already been observed in alpha waves in schizophrenia; alpha waves are collected in the EEG exam with closed eyes at rest but not sleeping, lying down, normal respiration, and in a calm environment (no auditory stimuli) [35]. It has been observed that the frequency is slower for people with schizophrenia, which impairs the process of perception and summarization of momentary circumstances. This supports the idea that people with schizophrenia may not perceive situations the same way as healthy people, leading to confusion in relationships due to misinterpretation [36,37,38]. Interhemispheric asymmetry (in size) is another difference that has been found in schizophrenia [39,40], which results in velocity differences for each hemisphere, causing cognitive problems (mental confusion, and this is one of the characteristics of schizophrenia) and motor system impairment. Low power in alpha waves has been found in many studies in schizophrenia. It is important to compare electrophysiological levels, which produce the electrophysiological parameters (voltage and frequency), of brains with schizophrenia with those of a healthy brain to measure the differences and make comparisons [41,42].

When individuals perform tasks, alpha waves are a good choice, given that the learning process occurs within this frequency range [41,42]. Therefore, they represent the best option for comparing these parameters between healthy individuals and those with schizophrenia with positive symptoms. Important information about mental effort is provided by the stability of electrophysiological levels, revealing fragility in electrolyte regulation. Not forgetting that a brain with schizophrenia (all types) is more sensitive to low glucose than a healthy brain [43,44]. Based on all this, what permits the comparison is a set of standardized tasks applied equally to both brains (healthy and unhealthy) in the study. The tasks must have the same cognitive load to make a fair comparison according to school-level equality [45,46].

So, this study aimed to investigate the use of electroencephalography (EEG) combined with standardized mental effort as a complementary tool for clinical diagnosis and to monitor treatment responses in individuals with schizophrenia and in those who wish to return to professional activities. To this end, voltage and frequency parameters of alpha waves in EEG signals—collected before and after a series of standardized and timed mental effort tasks—were compared between individuals with schizophrenia and a healthy control group. The durations of sustained mental effort were compared between the groups.

## 2. Materials and Methods

This is a descriptive and experimental study with a quantitative approach. Ethical approval was obtained from the Research Ethics Committee for Human Beings of the Instituto de Neurologia de Curitiba (INC), under opinion number 5.645.442/2023, and from the CONEP (National Research Ethics Commission) under opinion number 5.874.346/2023. The research team included the principal investigator (an electrical engineer with a master’s degree in biomedical engineering and specialization in EEG), a neurologist (EEG specialist), two psychiatrists, a biomedical scientist (EEG specialist), a psychologist (schizophrenia specialist), and a supervising professor.

All participants with schizophrenia were clinically stable (emotional and behavioral) and undergoing pharmacological treatment appropriate to their condition. Medication monitoring was not part of this study. All participants signed the Informed Consent Form. The conduct of this study follows the Declaration of Helsinki.

The researchers conducted all aspects of the experiment, including screening healthy volunteers, administering the tests (performed by the psychologist), and collecting data. Volunteers with schizophrenia were selected by the hospital’s psychiatry team. The study was conducted within the hospital for both groups (in different hospitals for each group); and for the schizophrenia group, a team of nurses and caregivers was available.

**Figure 1:**
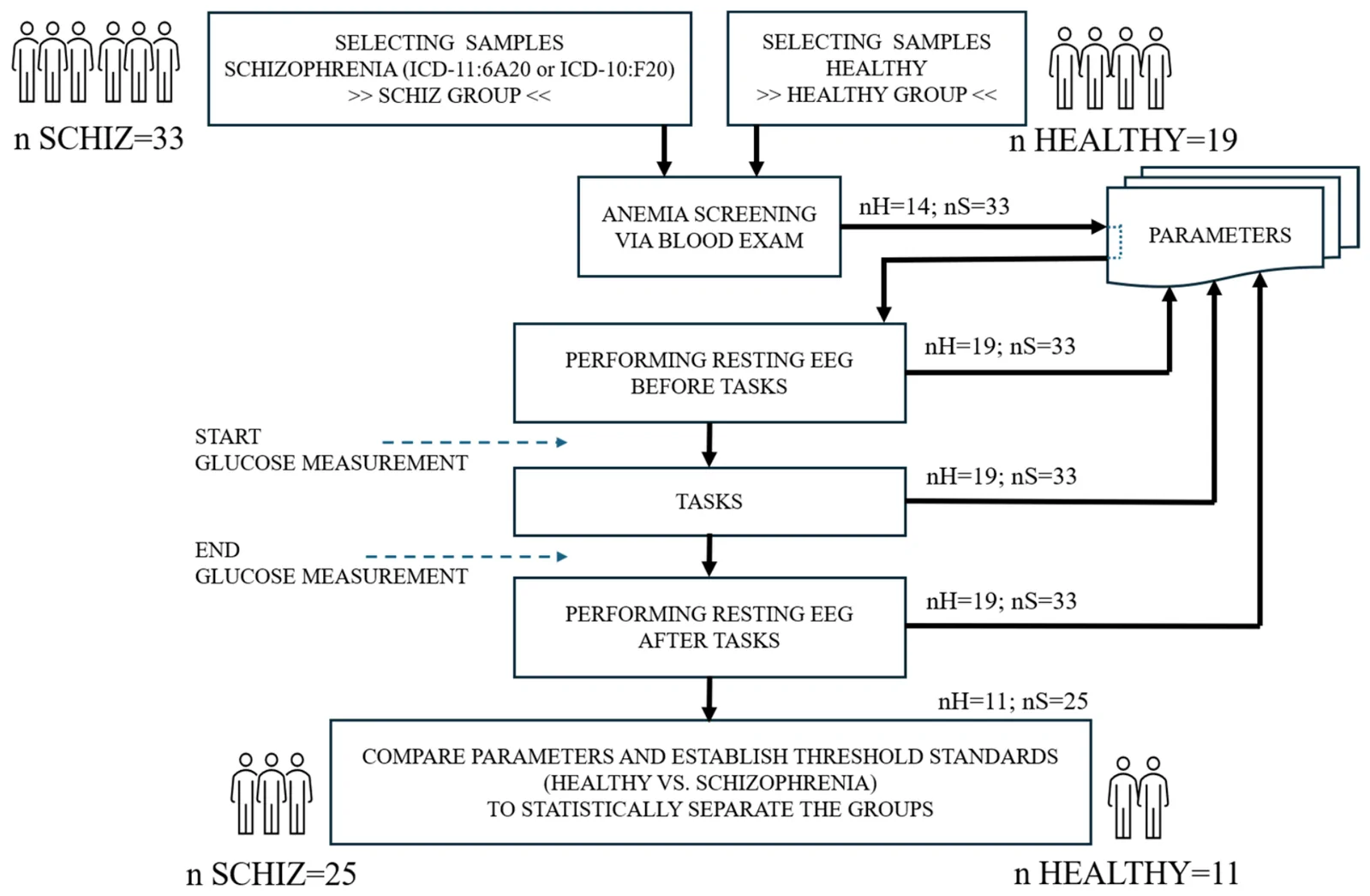
Research’s functional diagram indicating actions and parameters.

Study design:

EEG recordings were obtained from two groups of volunteers: a control group and a group with schizophrenia with positive symptoms. Neither group had anemia. Recordings were made before and after the performance of a standardized, timed sequence of tasks. Subsequently, voltage and frequency levels of the collected signals were calculated and compared between the groups, as was the time each volunteer took to complete each task (with the total time being the sum of the times for each task), as shown in Figure 1.

Before the EEG with mental effort exam, volunteers underwent blood tests to screen for anemia, and volunteers with anemia were excluded before the mental effort tests.

This study was divided into nine steps:

(1) Determining the minimum sample size;
(2) Recruiting volunteers according to the criteria (first screening);
(3) Conducting blood tests (second screening); iron-deficiency anemia was eliminated;
(4) Conducting electroencephalograms (EEGs) before performing the mental effort tasks (in IR phase);
(5) Measuring blood glucose levels before performing the mental effort tasks (third screening);
(6) Performing the mental effort tasks; timing (fourth screening);
(7) Measuring blood glucose levels after performing the mental effort tasks (fifth screening) (in FR phase);
(8) Performing the EEG after performing the mental effort tasks;
(9) Evaluating all research parameters.

Samples were excluded if they had anemia; blood glucose < 80 mg/dL (4.4 mmol/L) before or after tasks; did not complete all types of tasks; or did not show slow rest waves in IR or FR phases.

### 2.1. Sample Size

The sample size was calculated using the following equation, and the parameters were:

*n* = minimum number of samples;

*Z* = Z-score (a value from the normal distribution associated with the desired confidence level; for 95% confidence, Z = 1.96);

*p* = estimated proportion of the characteristic within the population (using a 99/1 ratio), since the prevalence of schizophrenia in the population is 1% (literature);

*N* = population under analysis (Curitiba has 1,832,183 people as of 2026);

*E* = tax error (5%).

(1) $$n=\frac{\frac{Z^{2}*\;p\;*\;(1-p)}{E^{2}}}{1+\frac{Z^{2}*\;p\;*\;(1-p)}{{N\;*\;E}^{2}}}$$

The result was *n* = 15.

Thirty-three participants with schizophrenia with positive symptoms who were clinically stabilized formed the SCHIZ group. Nineteen healthy individuals formed the HEALTHY control group.

### 2.2. Research Participants

Volunteers were recruited/selected based on the research’s clearly defined inclusion and exclusion criteria.

The general inclusion criteria were as follows: male sex, age 25–45, no ethnic or socioeconomic restrictions, normal blood tests, at least four years of formal education, clinical and emotional stability (no anxiety or agitation), and informed, voluntary consent.

The general exclusion criteria were as follows: presence of hyperactive behavior or inability to remain still during EEG recording; history of substance abuse (alcohol and/or drugs); social consumption of alcohol within 10 days before EEG or blood testing; presence of headache on the day of testing; diagnosis of uncontrolled diabetes mellitus; epilepsy; presence of an implanted cardiac pacemaker, artificial heart valves, or cerebrospinal fluid shunt devices.

Specific inclusion criteria for the schizophrenia group included a confirmed clinical diagnosis of schizophrenia with positive symptoms, preserved cognitive capacity, and stabilized clinical state.

Specific inclusion criteria for the HEALTHY group were based on preserved mental health.

Exclusion criteria for the HEALTHY group included abrupt mood swings, emotional instability, social or affective withdrawal, disordered appetite regulation, irregular sleep-wake cycles, cognitive impairments, panic disorder, depression, bipolar disorder, hyperactivity or restlessness, and use of psychiatric, sedative, or stimulant medications.

Two groups were included in the in vivo phase of the study: a clinical group composed of individuals with schizophrenia with positive symptoms and a matched control group of neurologically healthy participants.

Volunteers with schizophrenia were recruited from the psychiatric hospital in the metropolitan region of Curitiba, Brazil, chosen and registered to participate in the project registered on the Brazil Platform. The volunteers were selected and evaluated by the hospital team under the responsibility of the managing psychologist, also registered on the Brazil Platform. Of the 150 patients with schizophrenia who were hospitalized, only 33 met the requirements determined for the research.

Healthy participants were recruited primarily from the university (students or acquaintances) via internal dissemination of a digital recruitment form and enrolled if they met the criteria defined in the informed consent form for the control group. They were screened by the researcher. Participation was low due to the duration and complexity of the research.

All defined inclusion and exclusion criteria were included in the informed consent form. All research volunteers received detailed information about the objectives, procedures, and duration of the study before recruitment, before participating in the research, and again before signing the informed consent form.

### 2.3. Blood Exams

The blood test to check for anemia was performed by two laboratories (one accredited to the psychiatric hospital that collected samples within the hospital, and another external to the hospital, which tested healthy volunteers), but the clinical analysis methods were the same.

### 2.4. Glucose Dosage Before/After Tasks of Mental Effort

Capillary glucose measurements were performed on the first 10 volunteers in each group, before and after the mental exertion phase, using a portable glucometer, to ensure that blood glucose remained above 80 mg/dL (4.4 mmol/L). This precaution was taken due to the group with schizophrenia, given the known association between hypoglycemia and an increased risk of aggressive behavior in individuals with schizophrenia or fainting [43,44,47].

### 2.5. Medications

The volunteers with schizophrenia were in treatment with first, second, and third generation medications at varying dosages, administered in the morning (by 10:00 a.m.) and in the afternoon (after 05:00 p.m.). The EEG examinations involving mental effort were conducted at 3:00 p.m. (five hours after the last medication dose).

The effects of the medications on brain waves were not considered in this study, as this fell outside its scope (such information appeared as research data in the individual notes for each volunteer).

The Informed Consent Form did not mandate control over the medications; however, it did record the expected outcomes—namely, stabilized behavior and stable emotions.

### 2.6. EEG Routine Collection

All volunteers had general physical health within standard laboratory reference limits and were eligible for EEG acquisition according to the exclusion criteria.

Participants were instructed to maintain a normal sleep routine, avoid stimulants such as chocolate, coffee, and energy drinks, and consume their last meal at least 4 h before testing. EEGs were recorded using the International 10–20 System in accordance with the standards of the Brazilian Society of Clinical Neurophysiology and recorded in all positions of this configuration. Recordings were acquired for both groups under identical conditions. A1 and A2 were the reference points, and the ground was at the Fp position.

Data were collected in two phases: pre- and post-mental effort, that is, in the IR phase and in the FR phase. In both phases, participants remained in the supine position with eyes closed, awake, and relaxed. The protocol for EEG acquisition followed this sequence:

- Start of recording.
- Supine position.
- Initial Resting State (IR): eyes closed, relaxed, spontaneous breathing:
  - IR signal acquisition: 420 s.
- Initial alternating eyes open/closed: 30 s each, repeated 3 times.
- Hyperventilation phase 1 and recovery.
- Photic stimulation phase 1 (eyes closed) and rest.
- Verbal explanation of the task and rest procedures.
- Transition to seated position.
  - IR signal acquisition: 420 s.
- Initiation of mental effort task and instructions:
  - Activity 1, verbal fluency, 120 s; 300 s of rest;
  - Explanation of the next activity;
  - Activity 2, concentrated attention, 240 s; 300 s of rest;
  - Explanation of the next activity;
  - Activity 3, dispersive attention, 90 s; 300 s of rest;
  - Explanation of the next activity;
  - Activity 4, faces memorization, 240 s; 300 s of rest;
  - Explanation of the next activity;
  - Activity 5, faces recognition, 360 s; 300 s of rest;
  - Explanation of the next activity;
  - Activity 6, rules and patterns recognition, 1200 s; 300 s of rest;
  - Explanation of the next activity;
  - Activity 7, numerical reasoning, 1080 s; 300 s of rest;
  - Explanation of the next activity;
  - Activity 8, composition of colored figures, 900 s; 300 s of rest;
  - Explanation of the next activity;
  - Activity 9, musical sensation, 270 s; 300 s of rest;
  - Explanation of next activities.
- Return to supine position.
- Photic stimulation phase 2 and rest.
- Hyperventilation phase 2 and recovery.
- Final alternating eyes open/closed: 30 s each, repeated 3 times
- Final Resting State (FR): eyes closed, relaxed, spontaneous breathing:
  - FR signal acquisition: 420 s.
- End of recording.

### 2.7. Equipment and Signal Acquisition

EEG data were recorded using Neurotec equipment (model Neuromap EQSA260 40i—Manufacturer Neurotec, Itajubá 37504-109, Brazil), with a 22-channel acquisition system equipped with digital EEG and brain mapping software, and an 8-bit microprocessor, manufactured in Brazil. The system included photo- and audio-stimulation hardware with LED-based microprocessors. The notch filter was 60 Hz. No other filters were applied. There was a grounded electrical outlet.

Recordings were obtained using shell-type AgCl-coated electrodes in a double-banana montage, with 19 channels and impedances maintained below 5 kΩ. Signal amplitude (µV_micro-Volt) was recorded over time (s) at 256 samples/s and visualized using Neurotec software (version 3.7.3 and export module 1.2.55) on a laptop.

### 2.8. EEG Descriptors

EEG signals were analyzed in the time and frequency domains. Time domain descriptors included peak-to-peak (Vpp or Vt), maximum (Vmax), minimum (Vmin), average (Vmean), RMS (Vrms or Vr) voltage amplitudes, and frequency (f).

In the frequency domain, also called Spectral Analysis, it is possible to evaluate all frequencies that exist in that epoch and the power of each one.

**Figure 2:**
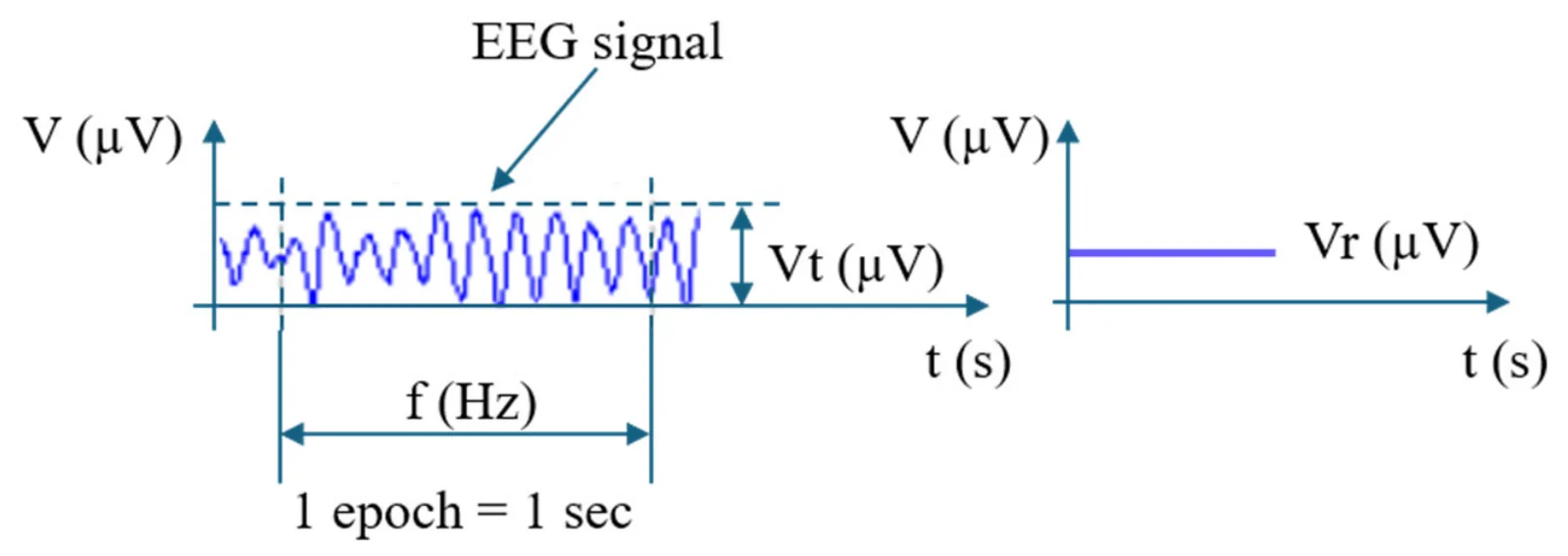
Descriptors in the time domain of the EEG signal (one second of the capture at the T6 point, for example): voltage (Vt and Vr) and frequency (f).

This study explored three descriptors, which are shown in Figure 2.

- Vpp or Vt (peak-to-peak voltage or total voltage amplitude or full amplitude), which reflects the maximum electrical potential of the brain (the sum of the electrolyte potentials), is the value of direct measurement by the EEG equipment, whose unit is microvolts (µV);
- Vr or Vrms (effective voltage or DC equivalent of Vt), which is the usual parameter for comparison between electrical systems, is calculated from Vt and is also referred to as the root-mean-square value. Mathematically, the RMS (Root Mean Square) value is the square root of the average of the squares of the values of a quantity at that moment. It is the equivalent value in the continuous current (DC) that dissipates the same power, as can be calculated by the following two equations:

(2) $$Vrms=\sqrt{\frac{1}{Ns}\sum_{i=1}^{Ns}V_{i}^{2}}$$

(3) $$Vrms=\sqrt{\frac{{V1}^{2}+{V2}^{2}+{V3}^{2\;}+\cdots +{V255}^{2}+{V256}^{2\;}}{256}}$$

$V_{i}^{2}$ value of the voltage at each sampling point;

Ns number of samples;

✓ In this study, each epoch was sampled 256 times, then Ns=256;
✓ It used 10 epochs in the IR phase and 10 epochs in the FR phase;
✓ Each epoch was 1 s.
✓ Remember that frequency (f) is the parameter registered in the time domain.

**Figure 3:**
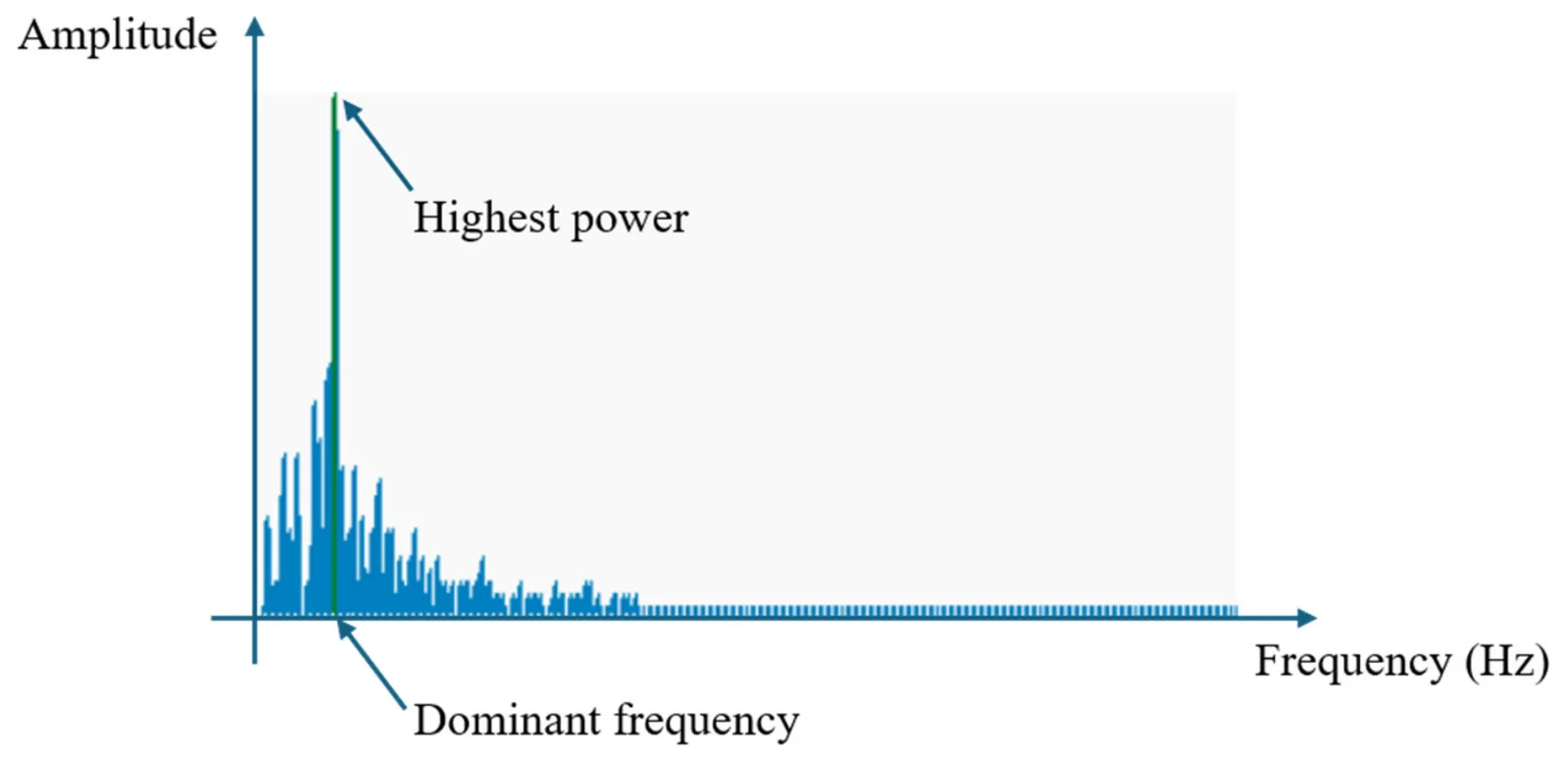
Descriptors in the frequency domain. In the frequency axis, all frequencies are shown by FFT; the peak frequency (highest) indicates the dominant frequency (F) in that epoch. The green line is a cursor that makes it easier to mark or find a specific frequency.

- In the Spectral Analysis, the dominant frequency (F) was chosen in each epoch. The dominant frequency (F), measured in hertz (Hz), is the frequency that has the highest power in the spectral analysis in each studied epoch, as shown in Figure 3. It was calculated for each epoch by FFT (FFT length = N = 256 = 2^8^). In the resulting frequency spectrum, the dominant frequency can be identified. Then, each epoch has a dominant frequency (F), and the result is the mean of all dominant frequencies (F) of each epoch.

All of these descriptors are analyzed at two phases in the EEG exam: at the beginning, Initial Register (IR), and at the end, Final Register (FR), that is, before and after the entire effort sequence.

Each epoch was sampled 256 times (2^8^ bits, the equipment’s microprocessor); Vr was then calculated as the average of the sum of the squares of the calculations performed on the 256 samples, and the spectral analysis contained 256 frequency samples.

Slow waves (sleep or waking in relaxation) have greater amplitude. When the brain is in an alert state (activity, stress, or anxiety), the amplitude is significantly lower and the frequency is noticeably higher.

### 2.9. Data Treatment and Analysis

For the EEG signals, data analysis was conducted with Neurotec (version 3.7.3), EDF (version 1.68 64 bits), MATLAB (version 2023a), Excel, and Minitab (version 17) software platforms. In this study, only clean epochs were selected, meaning that they contained no noise or artifacts across all EEG positions. This criterion was necessary to avoid the use of filters. As a precaution, the frequencies of eye blinking (2 Hz) and finger twitching (5 Hz) were recorded. However, only completely clean epochs were chosen, free of eye blinking or finger twitching.

Following this selection, the chosen epochs were identified individually. Each calculation was performed individually, and the results presented reflect the arithmetic mean of 10 epochs in IR and 10 epochs in FR; each epoch was 1 s.

Signal epochs were selected using Neurotec software and exported as EDF files. In the EDF software, the file was converted to CSV format for MATLAB-based signal processing. The results of each calculated epoch were compiled into an Excel spreadsheet, and the arithmetic mean (point-by-point) was computed. These results were then transferred to Minitab for statistical analysis. The Vt and Vr parameters were obtained using MATLAB’s toolboxes, and the dominant frequency was determined through Fast Fourier Transform (FFT) at each epoch, and it was considered the mean of them. Fast Fourier Transform (FFT) is an algorithm that calculates the Discrete Fourier Transform (DFT) of a sequence of sinusoids, allowing the conversion of signals from the time domain to the frequency domain. It decomposes a signal into sinusoidal components, allowing the identification of which frequencies compose the signal.

**Figure 4:**
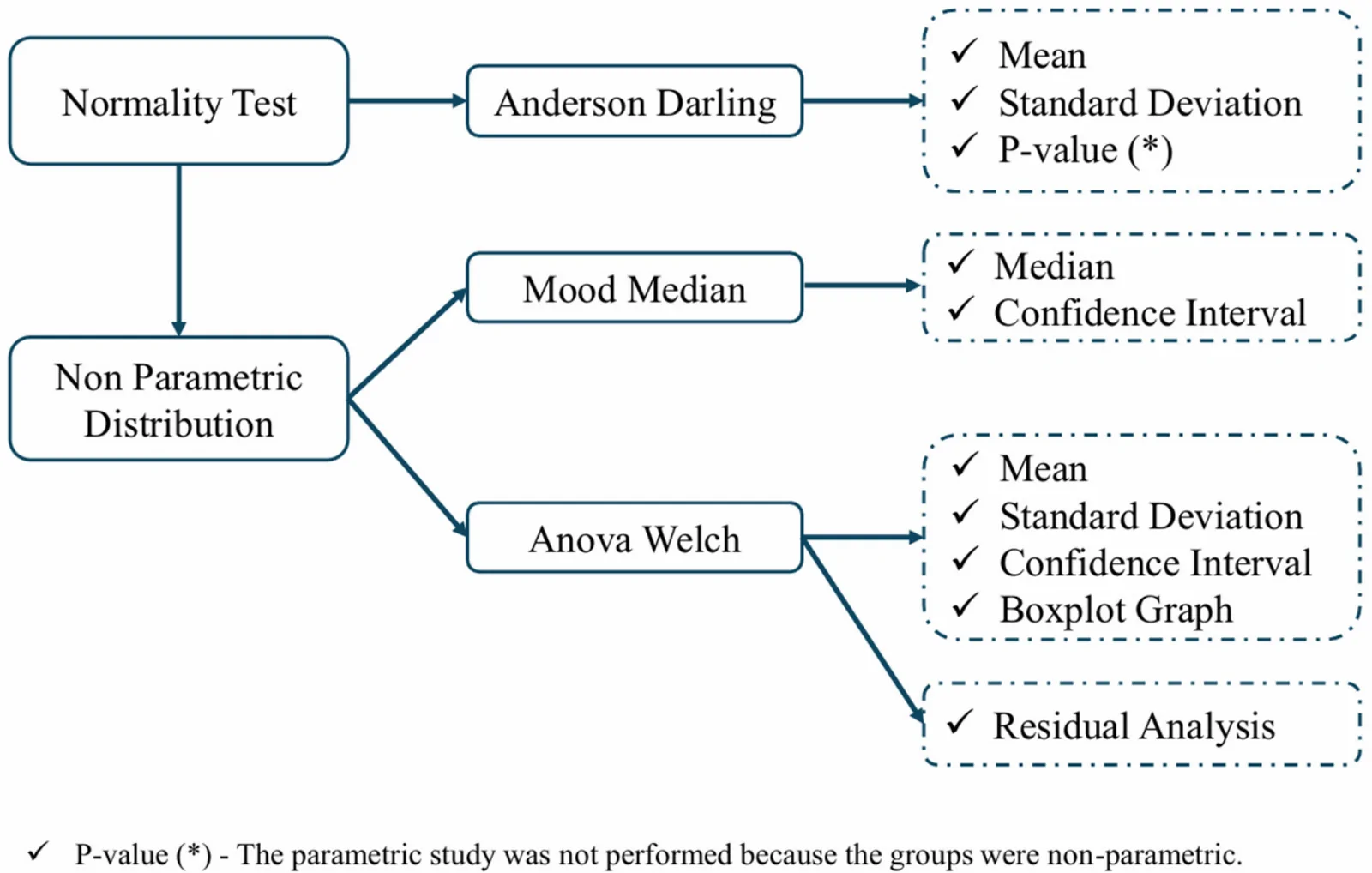
Statistical sequence of analysis of the EEG parameters.

For statistical analysis, the sequence shown in Figure 4 was followed.

Firstly, the Anderson–Darling normality test (α = 0.05) was applied. Then, any *p*-value > 0.05 was considered to indicate a normal distribution. The confidence level was 95%, as indicated in the sample size calculation and applied to all tests when necessary. Since the data distribution was outside the normal range (in the majority), the median was analyzed using Mood’s test (with confidence interval). To verify homoscedasticity, Welch’s ANOVA method was used. This method is appropriate for analyses with unequal variance and was used to check the means and obtain their confidence intervals and to create a box plot graph.

### 2.10. Mental Effort Tasks and Duration and Temporalization

Since schizophrenia significantly affects the capacity for mental (cognitive) effort, often resulting in reduced motivation and willingness to perform tasks that require high cognitive effort, in this study, we compared the total duration of mental effort tolerated by participants in each group. The cognitive effort protocol consisted of nine sequential phases with a maximum duration of 75 min, each task ranging from 2 to 20 min (tasks adjusted based on school year level), interspersed with rest intervals (300 s).

The tasks were arranged as follows: first, the simpler, shorter ones, followed by the more difficult, longer ones. The last two—creating colored figures and music sensation—were relaxation activities. The tasks and temporization are described in Section 2.6.

The tasks were verbal reasoning, concentrated attention, dispersive attention, face memorization, faces recognition, numerical reasoning, rules and pattern recognition, figure completion with colored pieces, and music sensation. All tests are accredited by the Regional Council of Psychology (CRP-Paraná/Brazil). All tasks (except the musical auditory perception task) were performed on appropriate paper.

The mental effort material, as well as details about it (authors, editions, and others), is the exclusive property of the professional psychology class and cannot be disclosed. Because of this, this research must be carried out together with a psychological professional. Each task was timed, but there was no pressure. The duration of each task was communicated to the volunteer during the explanation of each one, as shown in the EEG sequence, Section 2.6.

## 3. Results

The experiment was realized by researchers and followed the methodology described. The screening criteria were followed. After blood tests were performed and showed that none of the volunteers had iron-deficiency anemia. The EEG recordings were realized and processed as described in Data Treatment and Analysis. All volunteers had glucose levels between 80 mg/dL and 106 mg/dL (4.44 mmol/L–5.88 mmol/L) before and after completing the tasks of mental effort. There were no smokers or diabetics in any of the groups.

EEG recordings were obtained from 19 healthy volunteers and 33 individuals with schizophrenia. The average participant age was 30.3 years in the HEALTHY group and 31.5 years in the SCHIZ group. The average educational attainment was 15.5 years for HEALTHY and 8.08 years for SCHIZ.

All participants completed the research. However, 36.84% of healthy samples and 36.36% of SCHIZ samples were excluded due to the absence of alpha activity (8.0–13.0 Hz), the study’s focus. Final comparisons were made between 11 healthy and 25 schizophrenia samples.

The EEG descriptors selected for this study were Vt and Vr, and the dominant frequency (F) was verified in spectral analysis for the IR and FR phases.

The number of standard relaxation epochs differed between the groups. Healthy volunteers showed a mean of 24 one-second epochs (14 in IR and 10 in FR), while volunteers with schizophrenia showed a mean of 32 one-second epochs (17 in IR and 15 in FR). It is evident that both groups were more relaxed at the beginning.

Ten epochs (with 1 s each) were selected in the IR phase and ten epochs (with 1 s each) in the FR phase in both groups, as described in the methodology for EEG Routine Collection (IR signal acquisition: 420 s/FR signal acquisition: 420 s). The epochs were free of noise and artifacts. No filters were applied.

Additionally, the time volunteers devoted to the mental effort tasks was noted.

### 3.1. Blood Analysis

There were no cases of anemia in any of the groups.

It is known that anemia causes mental fragility, making mental effort an ordeal.

### 3.2. Vtotal (Vt)—Initial Register (IR) and Final Register (FR)

The parameter Vt represents the sum of the positive and negative half cycles. In the study of voltages, total voltage (Vt) is the metric that best reflects the integrity of the neuronal ensemble.

All EEG examination positions (System 10–20) were analyzed. The Anderson–Darling normality test was applied to each EEG position. The *p*-values obtained are presented in Table 1. It was considered that *p* > 0.05 indicates a normal distribution (alpha significance level equal to 5%).

Analysis of the total voltage amplitude (Vt) in the initial recording phase (IR) showed that all EEG electrode positions in the HEALTHY group had *p*-values > 0.05, indicating a normal distribution and supporting the use of parametric tests. In the final recording phase (FR), position A2 did not meet this criterion, indicating that a parametric distribution is not appropriate. The SCHIZ group showed many positions outside normality in the IR phase, including F7, T5, Fp2, and T4. This pattern was most pronounced in the FR phase, in which Fp1, F7, T3, T5, O1, and Fp2 showed distributions outside the normal range. Left lateralization was evident, as reported in the literature.

The *p*-value was high, indicating greater homoscedasticity for the HEALTHY group than for the SCHIZ group for Vt in the IR phase. The same occurred in the FR phase. When the distribution is not normal, homoscedasticity is compromised, as it is not possible to make an inference about a population. In this case, the median may indicate a tendency. The medians were calculated using the methodology, and the difference between the intergroup medians is shown in Table 1. Differences between medians near 20% were observed for points Fp1, F7, C3, O1, F4, and A2 in the IR phase and T3, O1, F4, O2, and A2 in the FR phase.

**Figure 5:**
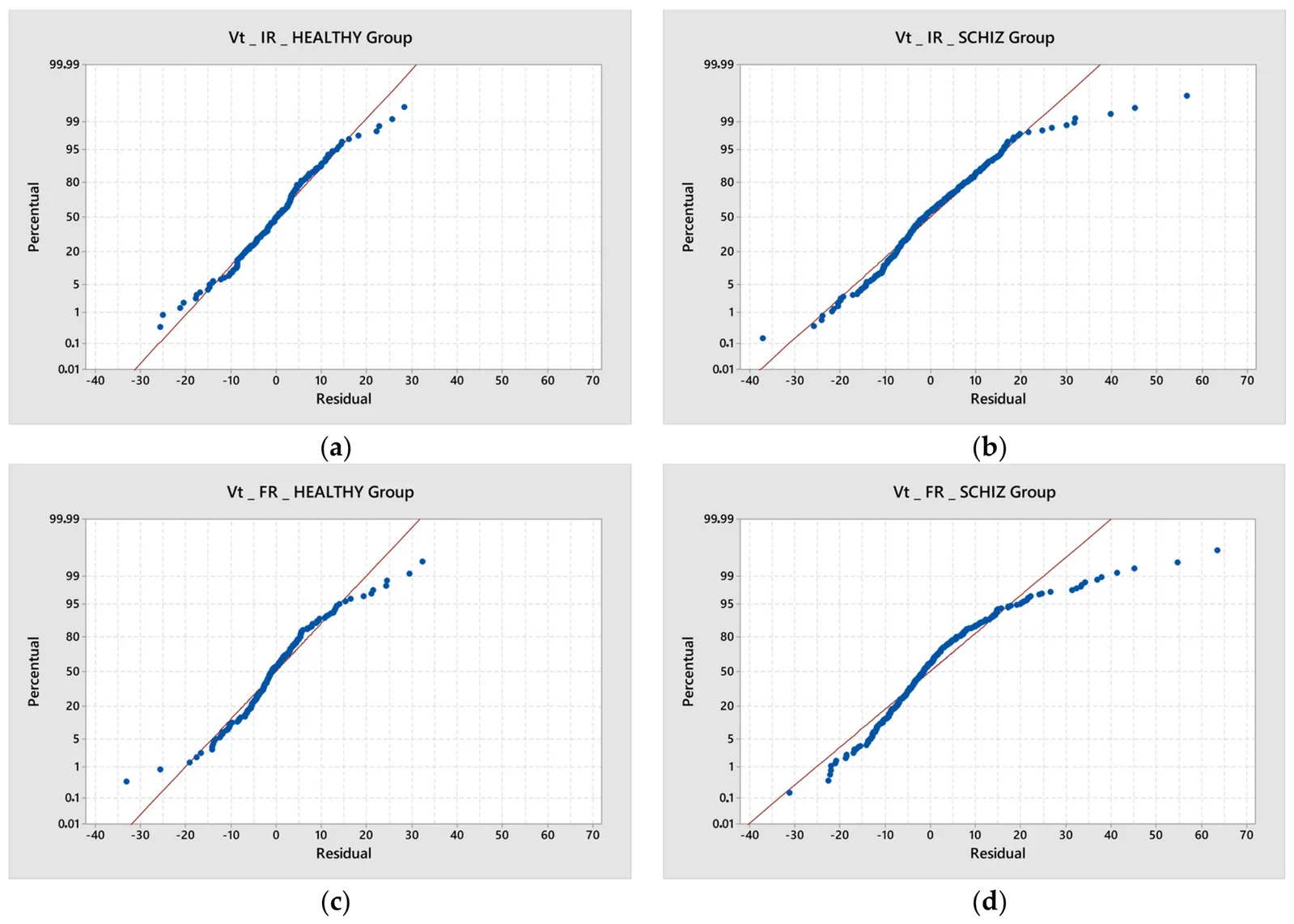
Residual analysis for Vt parameter: (**a**) IR phase for the HEALTHY group; (**b**) IR phase for the SCHIZ group; (**c**) FR phase for the HEALTHY group; (**d**) FR phase for the SCHIZ group.

Figure 5 shows the distribution of points in the homoscedasticity analysis. 

For all homoscedasticity diagrams, the red line represents the normal distribution of all residuals for each dataset under analysis. It indicates the ideal average behavior of the residuals for the defined confidence interval. In this article, each dataset (Vt, Vr, and F) is on the same scale to facilitate comparison between the groups in the IR and FR phases. The residuals of each dataset must be distributed over and along the entire red line, so this means that the confidence interval of my data is large. However, if the points are not over the red line, my confidence interval is reduced or interrupted. If the points are on the right side, the points are adjusted with a positive advantage. If the points are on the left side of the red line, then they are adjusted with a negative advantage. The further a point is from the red line, the less reliable that data point is (whether due to high variance or because it is an outlier).

For the Vt parameter in the IR phase, the homoscedasticity indicates a good fit in the same interval for both groups because the fit is adjusted. It was observed that the SCHIZ group exhibited better behavior than the HEALTHY group, as the points were positioned more precisely along the reference line. The intervals of the reliable residuals are between –7.5 to +15.0 for the HEALTHY group and –7.0 to +17.0 for the SCHIZ group in the IR phase. In the FR phase, –5.0 to +3.0 for HEALTHY group; and –6.0 to –3.0 and +13.0 to +16.0 for SCHIZ group (approximate data). 

However, in the FR phase, while the interval was reduced in the HEALTHY group, in the SCHIZ group, the interval practically disappeared. This indicates unstable behavior of the Vt parameter in relation to mental effort.

**Figure 6:**
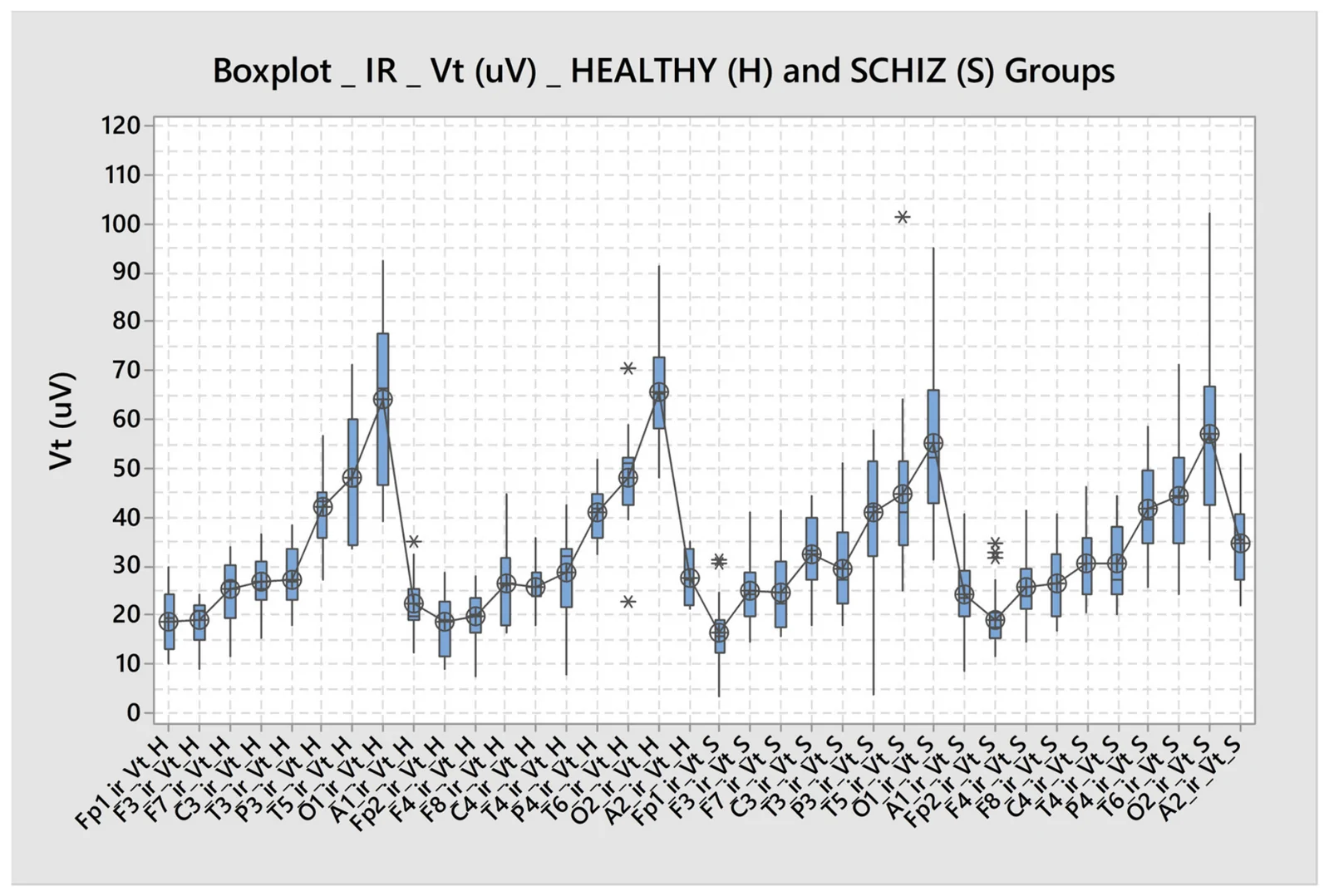
Comparison of the Vt (µV) parameter in IR phase between HEALTHY (H) and SCHIZ (S) groups. The nomenclature is: Position_Phase_Parameter_Group. On the left side of the graph is the HEALTHY (H) group, and on the right side is the SCHIZ group. Odd positions indicate the left hemisphere, and even positions indicate the right hemisphere.

**Figure 7:**
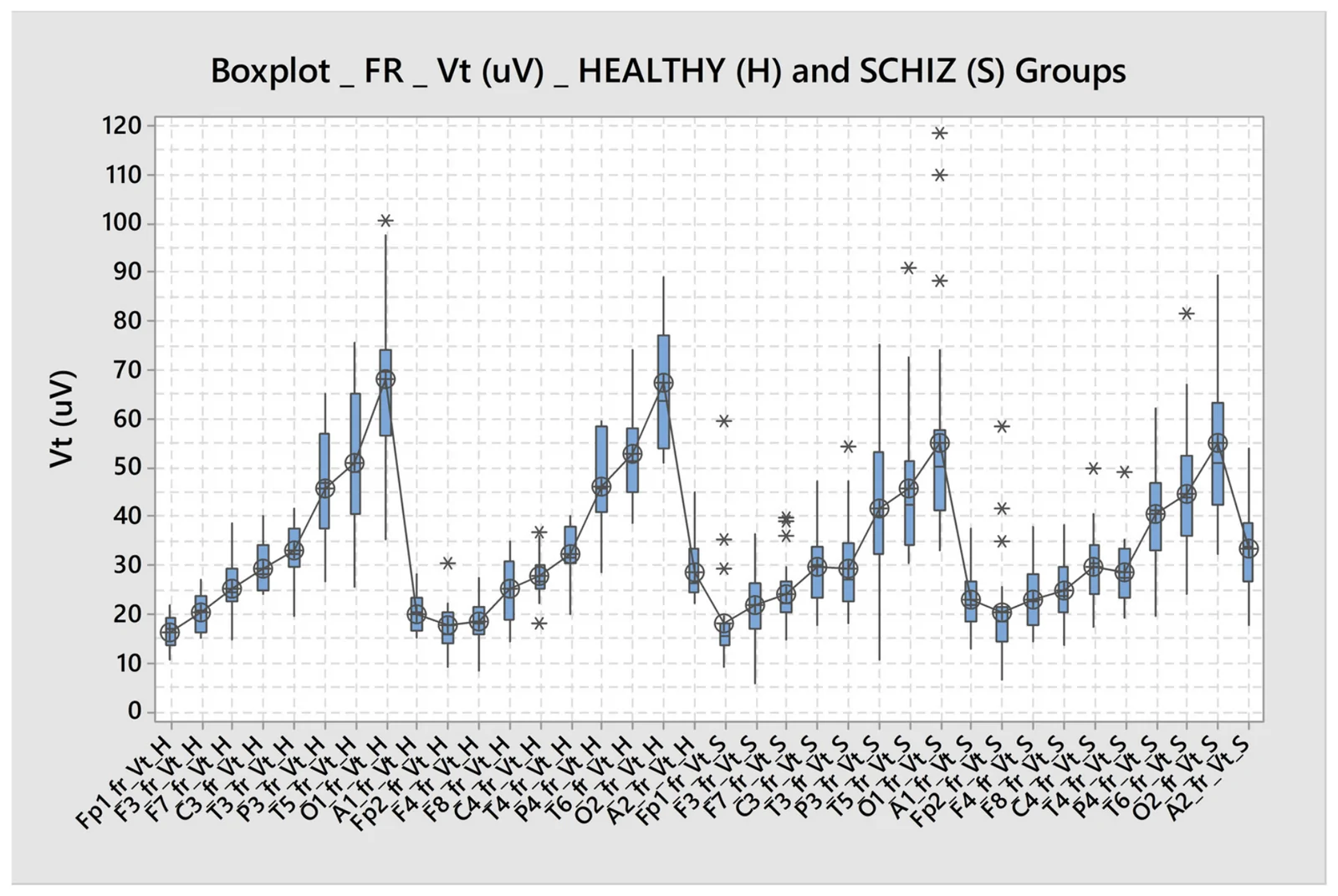
Comparison of the Vt (µV) parameter in FR phase between HEALTHY (H) and SCHIZ (S) groups. The nomenclature is: Position_Phase_Parameter_Group. On the left side of the graph is the HEALTHY (H) group, and on the right side is the SCHIZ group. Odd positions indicate the left hemisphere, and even positions indicate the right hemisphere.

Although it was not possible to make an inference about a population’s parameters due to heteroscedasticity, it was possible to verify a trend in the graphs generated by Welch’s ANOVA, in Figure 6 and Figure 7. The boxplot shows the distribution of parameter values. 

In these figures and in all other boxplot diagrams, a horizontal line running through the little box indicates the median value. Circles marked with a cross indicate the mean values. The line connecting the boxes joins the mean value of each little box. An asterisk-like symbol represents an outlier. The vertical lines extending from each box are the “whiskers,” which indicate the range and dispersion of the data.

Figure 6 shows the distribution of the Vt (µV) parameter values of both groups, and it is possible to perceive a harmonic pattern between them in the IR phase. Despite heteroscedasticity, a similar pattern of tracing was observed across all EEG points for both groups. However, long whiskers denote larger variance and greater disparity at that position in the brain. In the IR phase, few outliers were observed. The brains were at rest and balanced, but the SCHIZ group’s values were lower than those of the HEALTHY group.

In Figure 7, during the FR phase—that is, after mental effort—an important behavioral change was observed; many outliers indicated significant intra-group differences at that EEG position and, therefore, in the functioning of the cerebral lobes. Although the tracing was similar, the impression was of locking in disorganization in the SCHIZ group, suggesting that the effort makes the brain unbalanced. To analyze the tendency, the medians and the mean were calculated for comparison (because the mean is more sensitive to outliers). Significant differences were observed for O1 and O2 points in IR and FR phases for the SCHIZ group.

The median values for the Vt (µV) parameter in the IR phase for the HEALTHY group was 32.76 µV (mean 32.52 µV) for the left hemisphere and 34.20 µV (mean 33.42 µV) for the right hemisphere. In the FR phase, the medians were 34.35 µV (mean 34.41 µV) for the left hemisphere and 33.85 µV (mean 35.29 µV) for the right hemisphere. Resultant values were as folows: median 33.48 µV (mean 32.97 µV) in the IR phase, and median 34.10 µV (mean 34.85 µV) in the FR phase.

For the SCHIZ group, the medians were 31.18 µV (mean 32.47 µV) for the left hemisphere and 33.19 µV (mean 34.18 µV) for the right hemisphere in the IR phase. In the FR phase, the medians were 30.19 µV (mean 32.17 µV) for the left hemisphere and 32.63 µV (mean 33.40 µV) for the right hemisphere. Resultant values were as follows: median 32.19 µV (mean 33.33 µV) in the IR phase, and median 31.41 µV (mean 32.78 µV) in the FR phase.

In summary, globally, the median of the Vt (Vtotal) parameter for the SCHIZ group was 3.85% lower in the IR phase and 7.89% lower in the FR phase than that for the HEALTHY group.

### 3.3. Vrms (Vr)-Initial Register (IR) and Final Register (FR)

Vrms is the root mean square of the Vtotal (Vt); therefore, the calculated values should follow the same behavior as Vt. All calculated values were tabulated, and the statistical treatment was applied following the proposal explained in the methodology. All EEG examination positions (System 10–20) were analyzed. The Anderson–Darling normality test was applied to each EEG position, and the *p*-values obtained are listed in Table 2. A *p*-value greater than 0.05 was considered indicative of a normal distribution (alpha significance level of 5%).

The analysis of the Vrms (Vr) in the initial recording phase (IR) revealed that all EEG electrode positions in the HEALTHY group presented *p*-values greater than 0.05, indicating a normal distribution, which allows a parametric analysis to be possible. In the final recording phase (FR), many positions altered their behavior, including C3, F4, F8, C4, and A2, failing to maintain Vrms and yielding *p*-values less than 0.05 and rendering parametric analysis impossible. For the SCHIZ group, the positions that failed to maintain Vrms in the initial recording phase (IR) were Fp1, T5, A1, Fp2, F4, F8, and T4, and in the final recording phase (FR), Fp1, F3, T5, O1, A1, Fp2, F8, and O2. Due to the heteroscedasticity, it is not possible to make an inference about a population, but the median may indicate a tendency. The medians were calculated following the methodology, and differences between medians near 20% were observed for points Fp1, C3, O1, F4, O2, and A2 in the IR phase and for O1, C4, T4, T6, O2, and A2 in the FR phase.

**Figure 8:**
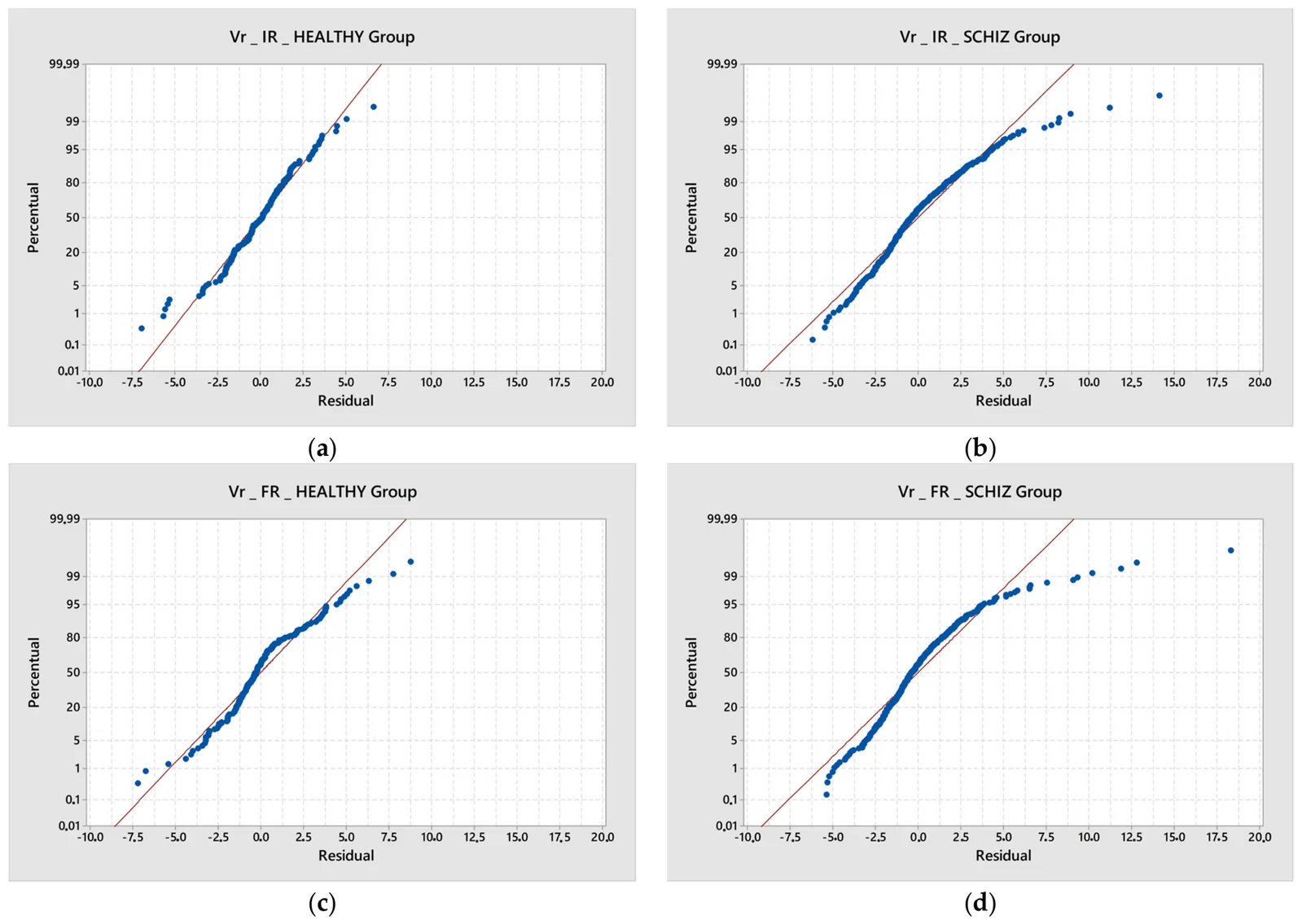
Residual analysis for the Vr parameter: (**a**) IR phase for the HEALTHY group; (**b**) IR phase for the SCHIZ group; (**c**) FR phase for the HEALTHY group; (**d**) FR phase for the SCHIZ group.

**Figure 9:**
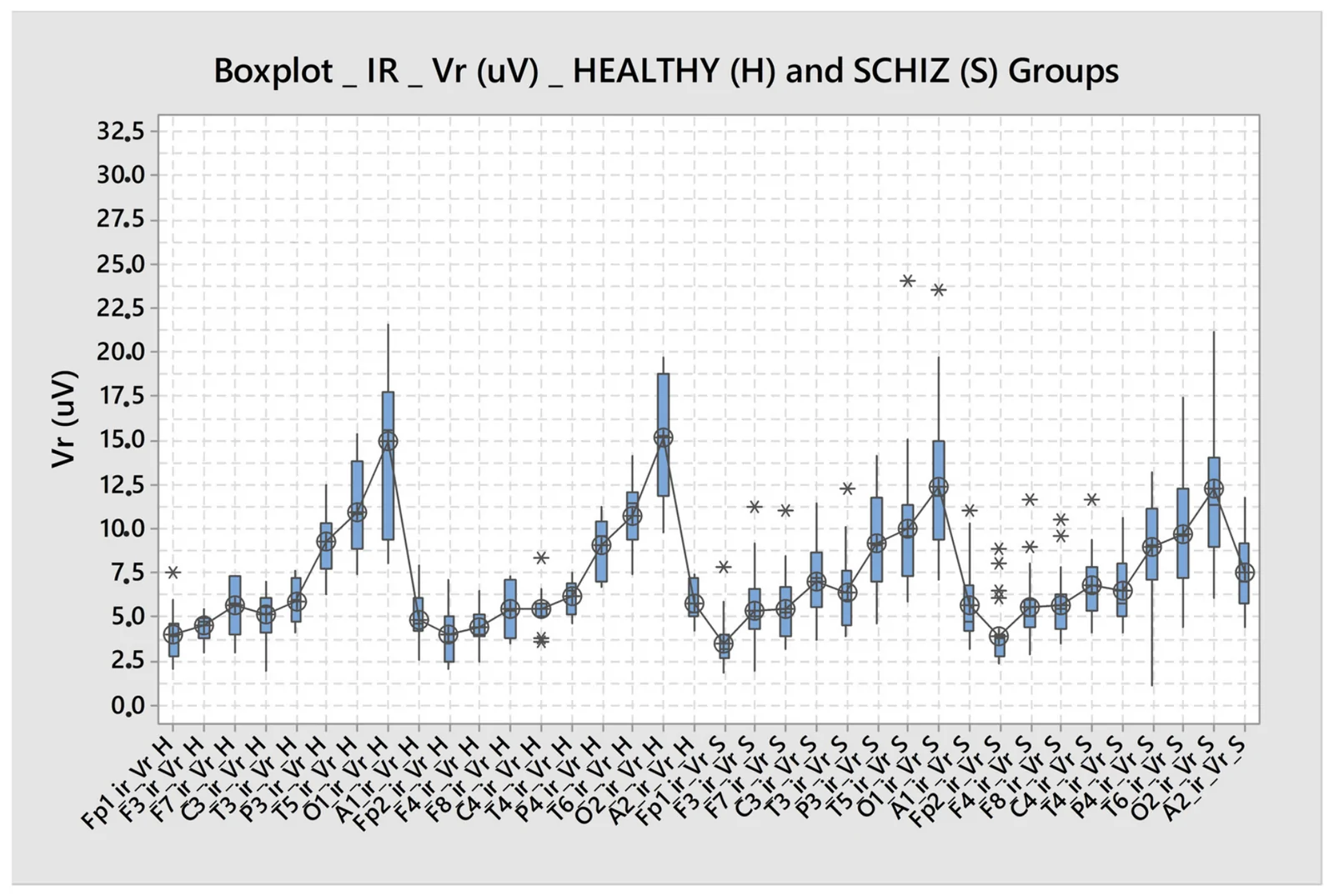
Comparison of the Vr (µV) parameter in IR phase between HEALTHY (H) and SCHIZ (S) groups. The nomenclature is: Position_Phase_Parameter_Group. On the left side of the graph is the HEALTHY (H) group, and on the right side is the SCHIZ group. Odd positions indicate the left hemisphere, and even positions indicate the right hemisphere.

**Figure 10:**
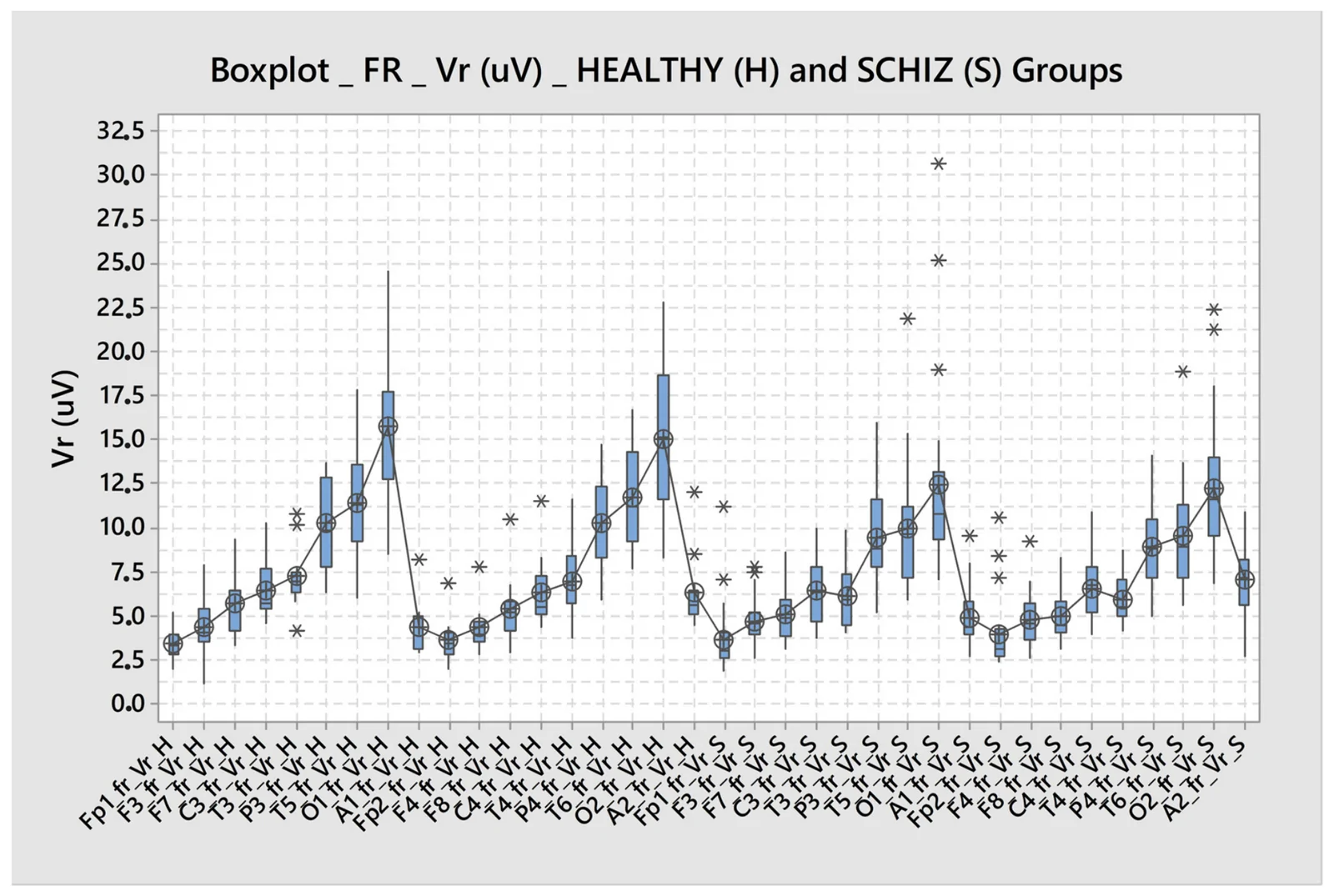
Comparison of the Vr (µV) parameter in FR phase between HEALTHY (H) and SCHIZ (S) groups. The nomenclature is: Position_Phase_Parameter_Group. On the left side of the graph is the HEALTHY (H) group, and on the right side is the SCHIZ group. Odd positions indicate the left hemisphere, and even positions indicate the right hemisphere.

Homoscedasticity followed the same pattern as Vt (due to its function), and it is possible to observe this in Figure 8. Vr parameter had good behavior in the homoscedasticity during the IR phase while the residuals are between ±2.0 for the HEALTHY group; and between to – 2.0 to 0 and +1.25 to +3.75 for the SCHIZ group. In the FR phase, the interval is so short; between –1.25 to –0.5; and +2.0 to +3.0 for the HEALTHY group. For the SCHIZ group between –2.0 to 0.0 and +3.0 to +4.0 (approximate data). This means high heteroscedasticity, and it is not possible to infer over the population but is possible to verify the tendency through the median, which is given in Figure 9 and Figure 10.

Welch’s ANOVA was used to create the boxplot. In the distribution of values of the Vr (uV) parameter, a few outliers were observed in the HEALTHY group in the IR phase, and many in the FR phase. For the SCHIZ group, there were many outliers in the IR phase, and the same for the FR phase. The trace was not so harmonic, which shows the differences between the functionality into brain areas. To analyze the tendency, the medians and the mean are calculated. The median values for the Vr (µV) parameter in the IR phase for the HEALTHY group were 7.34 µV (mean 7.23 µV) for the left hemisphere and 7.36 µV (mean 7.34 µV) for the right hemisphere. For the FR phase, the medians were 7.47 µV (mean 7.69 µV) for the left hemisphere and 7.45 µV (mean 7.73 µV) for the right hemisphere. Resultant values were as follows: median 7.35 µV (mean 7.29 µV) in the IR phase, and median 7.46 µV (mean 7.72 µV) in the FR phase.

For the SCHIZ group, the medians were 6.86 µV (mean 7.18µV) for the left hemisphere and 7.03 µV (mean 7.40 µV) for the right in the IR phase, and 6.52 µV (mean 7.00 µV) for the left hemisphere and 6.81 µV (mean 7.12 µV) for the right hemisphere in the FR phase. Resultant values were as follows: median 6.94 µV (mean 7.29 µV) in the IR phase, and median 6.66 µV (mean 7.06 µV) in the FR phase.

In summary, globally, the median of the Vr (Vrms-µV) parameter for the SCHIZ group was 5.55% lower in the IR phase and 10.70% lower in the FR phase than that for the HEALTHY group.

### 3.4. Spectral Analysis-Initial Register (IR) and Final Register (FR)

The analysis was performed at the same epoch as the voltage analysis. The analysis parameter was the dominant frequency (F) of each chosen epoch (10 distinct epochs of 1 s each). The dominant frequency is the frequency that presents the highest power during the analyzed period. It was obtained using the FFT (Fast Fourier Transform) for each analyzed epoch, and then, the average of these was calculated for each sample (research volunteer). No filter was applied because the chosen epochs were clean (the epochs were carefully chosen as described in the methodology). The results were tabulated, and statistical analyses were performed for all positions of the EEG signal in the IR and FR phases.

Initially, the Anderson–Darling normality test was applied to each EEG position. The *p*-values obtained are listed in Table 3. It was considered that a *p*-value greater than 0.05 indicates a normal distribution (alpha significance level of 5%), allowing a parametric analysis.

The analysis of the frequency (F) in the initial recording phase (IR) for the HEALTHY group revealed that all EEG electrode positions had *p*-values greater than 0.05, except C3, P3, A1, T6, and A2. In the FR phase, Fp1, F7, C3, T3, O1, A1, Fp2, F8, P4, T6, and A2 showed distributions outside normality. The frontal lobe showed some points outside normality after the mental effort. For the SCHIZ group, in the IR phase, Fp1, F3, F7, T3, A1, Fp2, C4, P4, and A2 showed distributions outside normality. In the FR phase, F7, C3, Fp2, F4, C4, T4, and T6 showed abnormal distributions. Therefore, parametric analysis was not possible for both groups.

Although it was not possible to infer information about a single population due to the distribution not falling within the normality parameters and the absence of homoscedasticity, a study was conducted to verify the tendency in the research data using the median. The Mood Median Method was used. It was possible to visualize the differences between medians, and the differences near 20% were observed for points C3, T3, P3, Fp2, F4, F8, P4, and T6 in the IR phase and Fp1, F3, F7, C3, T3, O1, Fp2, F4, F8, C4, and T4 in the FR phase.

**Figure 11:**
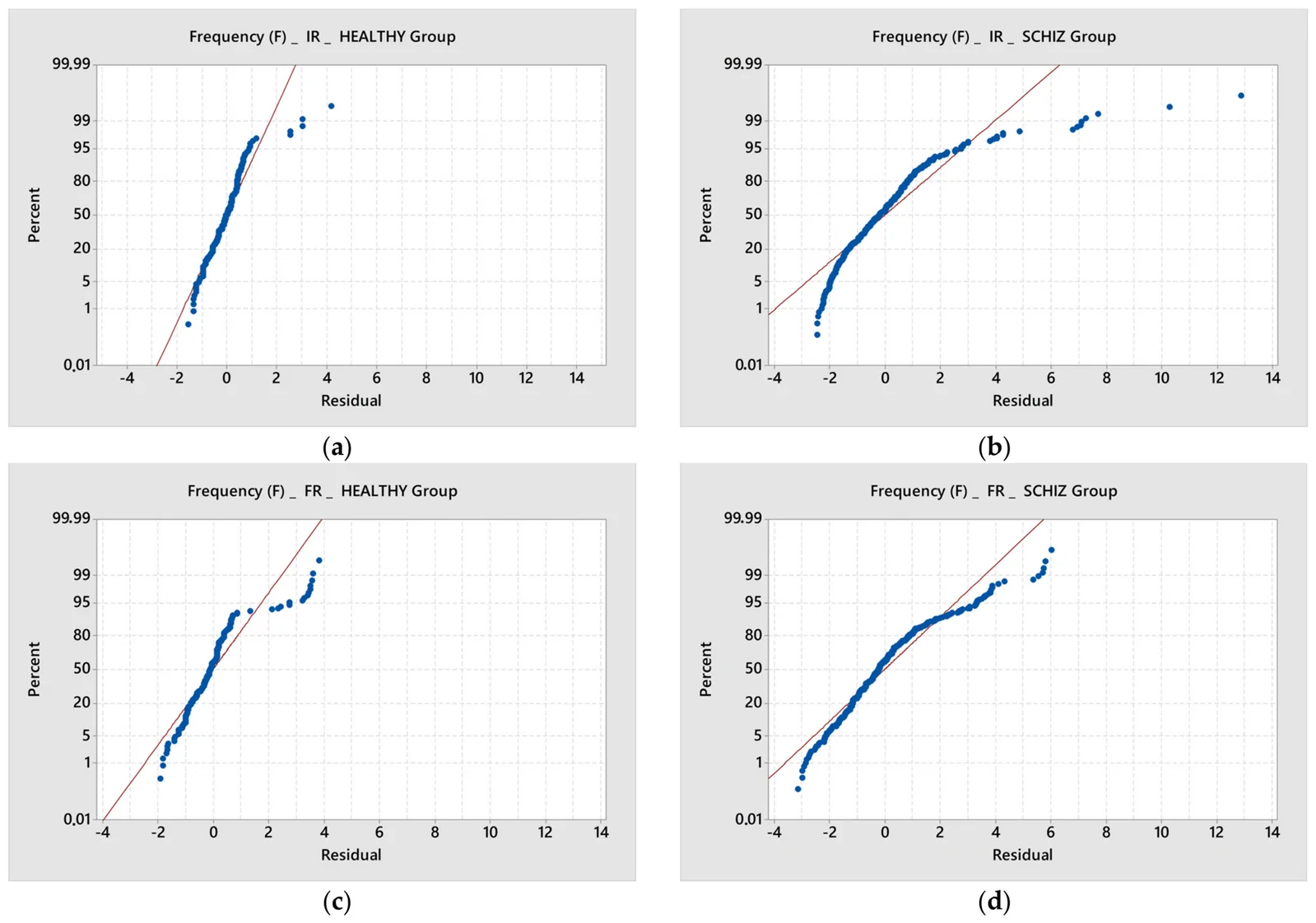
Residual analysis for F parameter: (**a**) IR phase for HEALTHY group; (**b**) IR phase for SCHIZ group; (**c**) FR phase for HEALTHY group; (**d**) FR phase for SCHIZ group.

The residual analysis indicates a good fit only in Figure 11a. Many residuals were positive and negative, and it was evident that the outliers significantly influenced the residuals in both groups. The intervals of reliable residuals are short: –1.0 to +0.5 for the HEALTHY group; –1.5 to 0.0 for the SCHIZ group in the IR phase. For the FR phase, –1.0 to +0.3 for the HEALTHY group and –1.0 to 0.0 and +1.8 to +2.2 for the SCHIZ group (approximate data). Many negative residual points indicate that the real value is lower than the value predicted by the reference line calculated based on the data of the determinant frequency for both groups. 

Homoscedasticity was present only in the HEALTHY group during the IR phase; it disappeared in the FR phase, given that only a small range of points fell on the reference line. In the SCHIZ group, heteroscedasticity was observed in both the IR and FR phases, with few points over the reference line. Due to this issue, it was impossible to make parametric inferences about the population. For the SCHIZ group, a misfit occurred, although it still maintained some proximity to the reference line.

**Figure 12:**
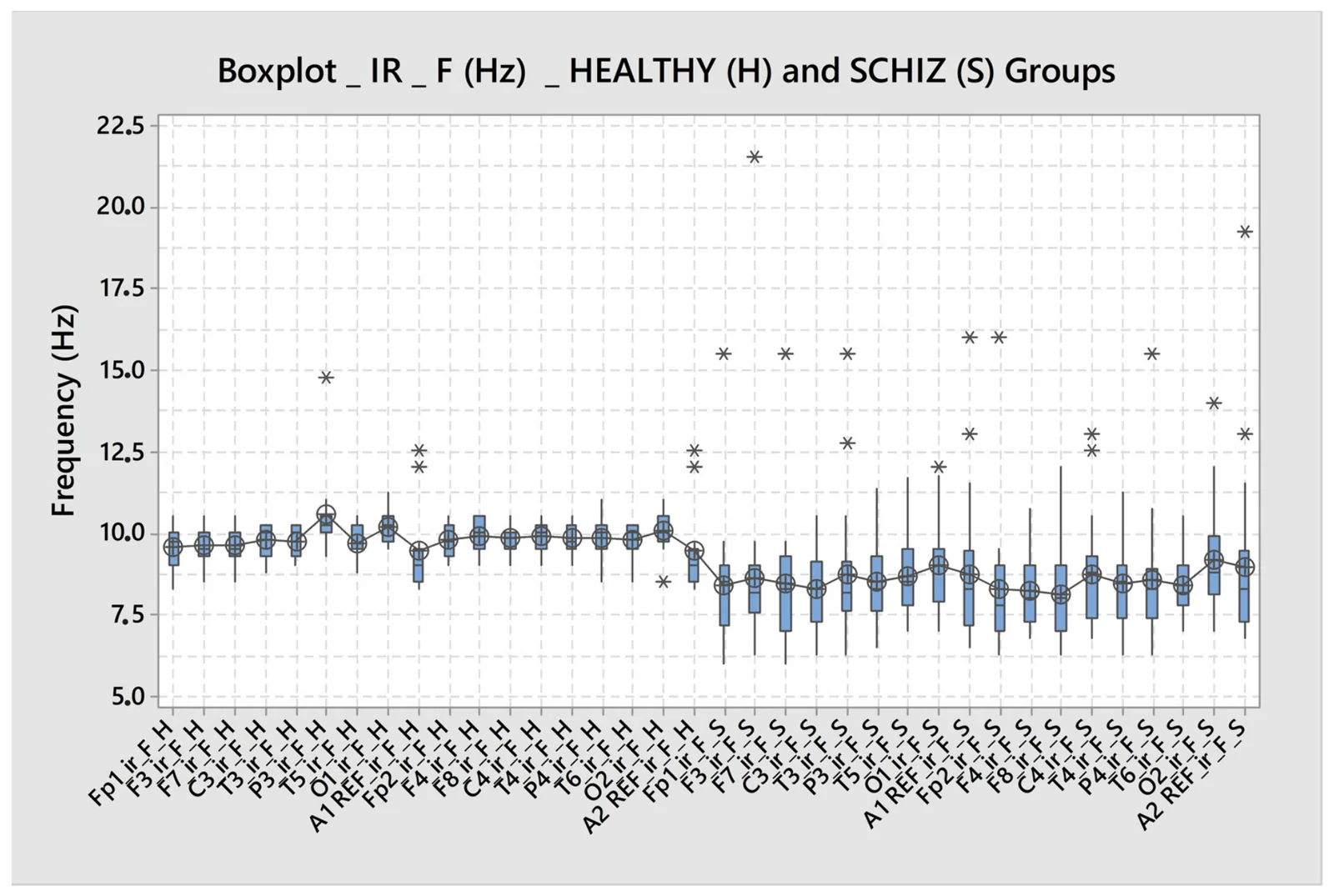
Comparison of the F (Hz) parameter in IR phase between HEALTHY (H) and SCHIZ (S) groups. The nomenclature is: Position_Phase_Parameter_Group. On the left side of the graph is the HEALTHY (H) group, and on the right side is the SCHIZ group. Odd positions indicate the left hemisphere, and even positions indicate the right hemisphere.

**Figure 13:**
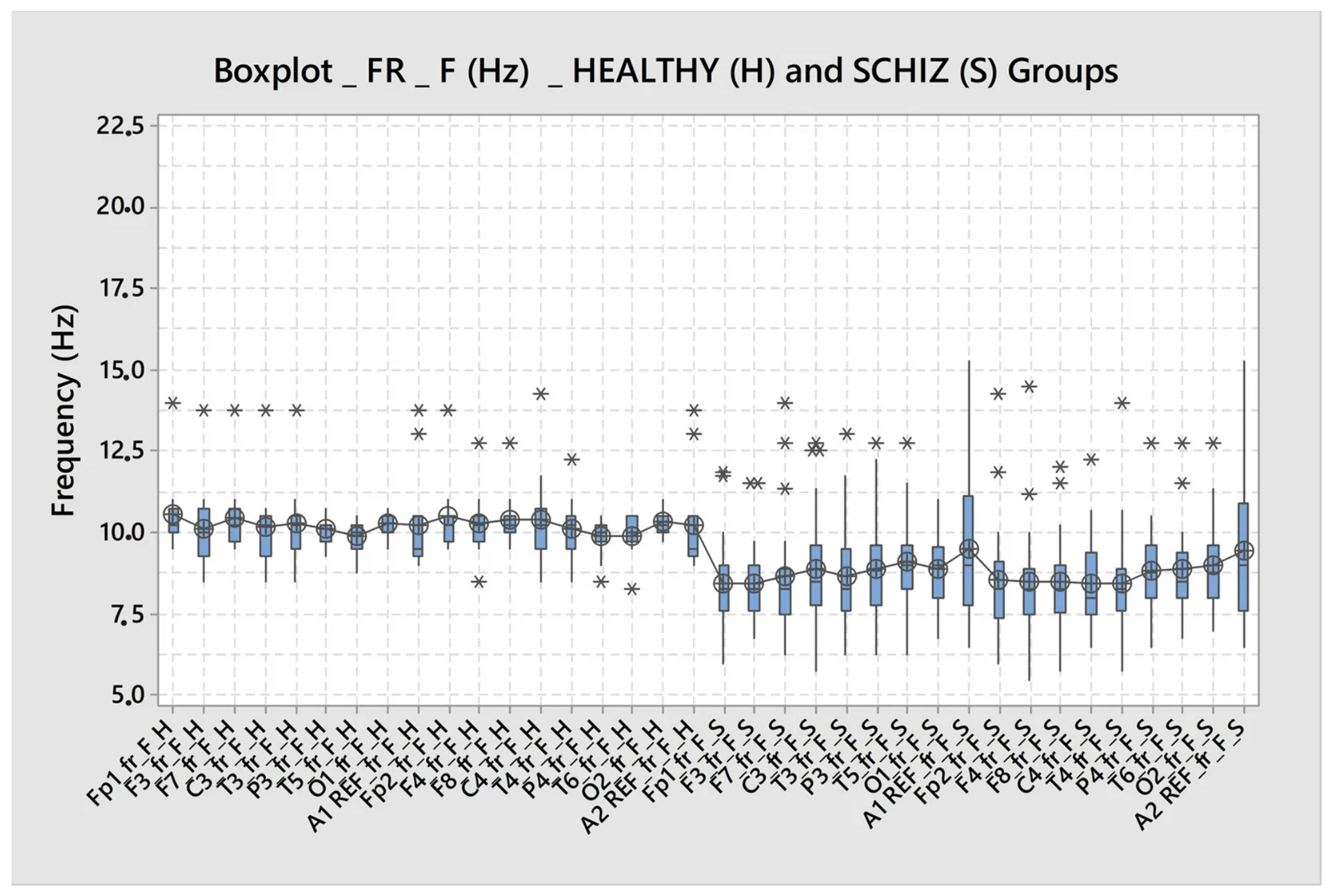
Comparison of the F (Hz) parameter in FR phase between HEALTHY (H) and SCHIZ (S) groups. The nomenclature is: Position_Phase_Parameter_Group. On the left side of the graph is the HEALTHY (H) group, and on the right side is the SCHIZ group. Odd positions indicate the left hemisphere, and even positions indicate the right hemisphere.

However, the tendency, Welch’s ANOVA, and the graphs in Figure 12 and Figure 13 show the distribution of the dominant frequency parameter values.

The frequency thresholds were distinct between groups. There was more pronounced rippling in the SCHIZ group, whereas in the HEALTHY group, it was soft. Graphically, the frequency distribution for the HEALTHY group was close to 10 Hz, with short boxplots and whiskers, indicating low dispersion. Note that the left and right hemispheres were like small undulations. In the SCHIZ group, larger undulations could be observed. The long boxplots with long whiskers indicated greater dispersion. Many outliers were also observed in the FR phase for both groups, with a significant increase in relation to the IR phase. To analyze the tendency, the medians and mean were calculated.

The median values for the F (Hz) parameter in the IR phase for the HEALTHY group were 9.72 Hz (mean 9.74 Hz) for the left hemisphere and 9.83 Hz (mean 9.74 Hz) for the right. For the FR phase, the median was 10.14 Hz (mean 10.24 Hz) for the left hemisphere and 10.11 Hz (mean 10.22 Hz) for the right hemisphere. Resultant values were as follows: median 9.78 Hz (mean 9.74 Hz) in the IR phase, and median 10.13 Hz (mean 10.23 Hz) in the FR phase for the HEALTHY group.

For the SCHIZ group, the median was 8.37 Hz (mean 8.57 Hz) for the left hemisphere and 8.27 Hz (mean 8.50 Hz) for the right hemisphere in the IR phase, and 8.50 Hz (mean 8.80 Hz) for the left hemisphere and 8.44 Hz (mean 8.75 Hz) for the right hemisphere in the FR phase. Resultant values were as follows: median 8.32 Hz (mean 8.54 Hz) in the IR phase, and median 8.47 Hz (mean 8.77 Hz) in the FR phase for the SCHIZ group.

In summary, globally, the F (frequency-Hz) parameter for the SCHIZ group was 14.91% lower in the IR phase and 16.35% lower in the FR phase than that in the HEALTHY group; that is, the frequency was lower in both phases for the SCHIZ group.

### 3.5. Time Effort

The mental effort tasks were administered according to each volunteer’s educational level. All volunteers completed the nine activities for the maximum time they could dedicate to each one, and the times were added together (the sum of all activities completed in full was 75 min, and it represented 100%).

All volunteers were encouraged to participate in the activities until completion, but despite their goodwill, not all completed each activity until the end.

The tasks with the highest dropout rates were rules and pattern recognition, numerical reasoning, and face recognition, in that order; the school environment was observed to influence this aspect, even though the tests were equivalent in terms of educational level. Volunteers in the SCHIZ group with higher levels of education persisted longer in their attempts (and responses) rather than giving up.

In the SCHIZ group, the following was observed: activities (1–3; 9), no cases of desistance; activities (4, 5, 8), many cases of fatigue; activities (6, 7); more cases of poor comprehension.

In the HEALTHY group, the following was observed: activities (1–3; 8, 9), no cases of desistance; activities (4, 5), many cases of fatigue; activities (6, 7); they thought that was interesting.

Activities (8) and (9) were aimed at relaxation but were not well received by all SCHIZ volunteers; activity (8–organization and focus function) made some volunteers impatient, and those same volunteers—who had shown impatience with activity (8)—also became impatient with the music activity (9–emotional regulation). The HEALTHY volunteers liked both activities.

For evaluation of the time of each volunteer, in all groups, when the volunteer finished the task earlier, it was considered 100%, but when the volunteer declined the task, it was considered partial.

For example:

Volunteer 1; task (5); (6 min duration); finished (did all questions) in 5 min; then it was considered 100%;

Volunteer 25; task (5); (6 min duration); gave up at 5 min (did not answer all questions); then it was considered 83.33%;

The final result (that is shown in the graph) is the meaning of the summatory.

The scores (whether the answer was corrected or not) are not considered in this study.

Motivation, anxiety, medication effects, drowsiness, fatigue, personal preference (liking or disliking a specific activity), and circadian rhythm are factors that can influence the results that remained uncontrolled.

**Figure 14:**
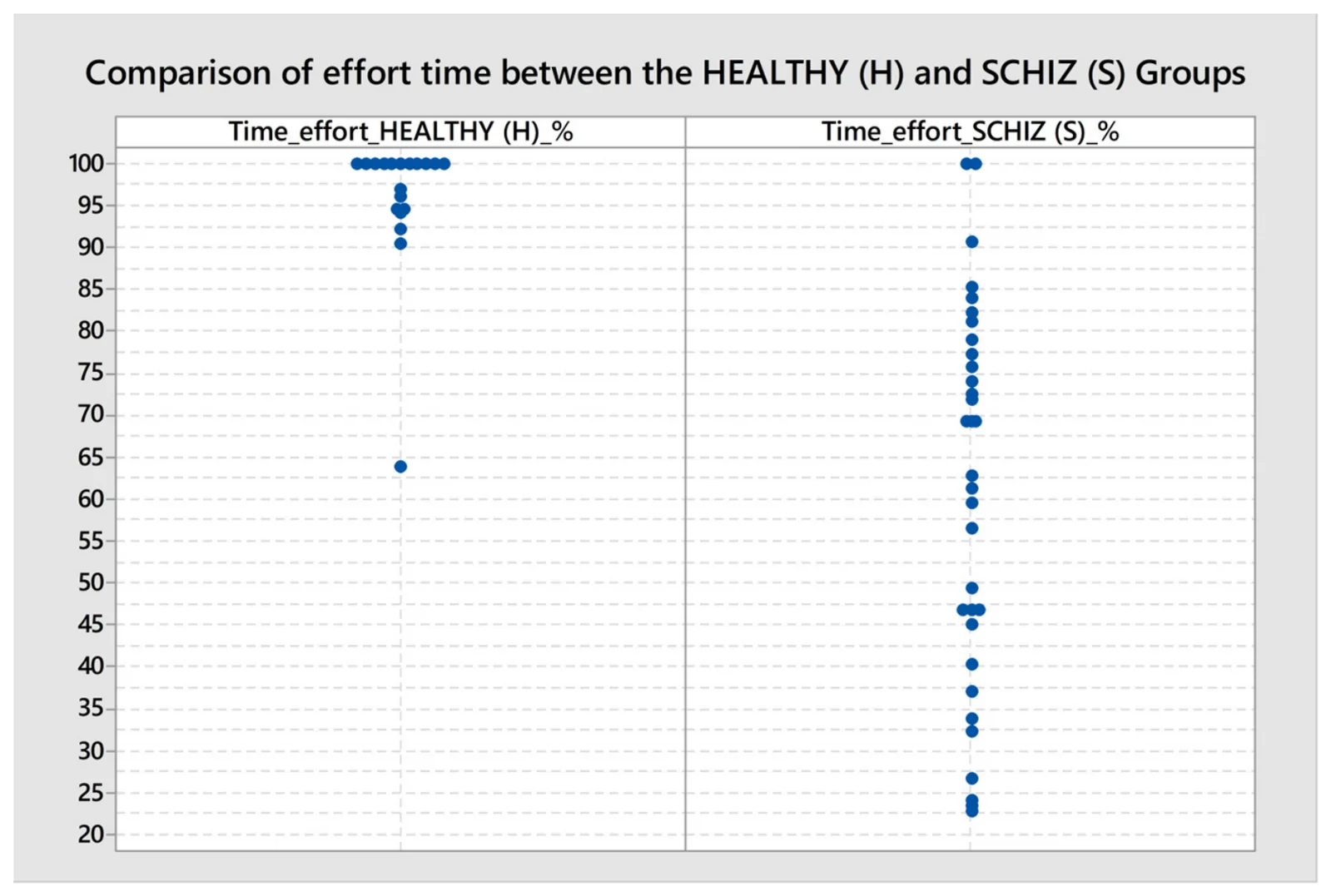
Comparison of the time supported under mental effort between the groups.

The graph in Figure 14 shows the time each research volunteer devoted to mental effort as a percentage (%). It shows the results of the 33 volunteers with schizophrenia (SCHIZ group) and 19 healthy volunteers (HEALTHY group). There were 11 results at 100% for the HEALTHY group (the rest are above 90%), and only one at 64% (*). For the SCHIZ group, 2 results were 100%, and the rest ranged from 22.66% to 90.66%. The difference between the groups was significant.

The case of the HEALTHY group with 64% (*) was under suspicion. Later, the volunteer was confirmed to have schizophrenia (and was excluded from the healthy group and was not included in the schizophrenia group in this study).

## 4. Discussion

Schizophrenia does not always arrive slowly in adolescence. It can, exceptionally, appear after very long periods of stressful work, poor sleep, an unhealthy lifestyle, drugs, poor nutrition, or after a brain injury, as stated by Jurgens and Johnson [48] and Cheng et al. [49]. Businessmen, engineers, doctors, and lawyers can also be affected. It does not necessarily appear only in people who were already performing poorly in school, as pointed out by Černis et al. [50]. Building on this, people with substantial intellectual capacity may be especially reluctant to leave their professional activities, given the significant personal investment involved. Nevertheless, if such professionals develop schizophrenia, they often lose their jobs because they can no longer maintain the same hours or workload. These questions are connected to the objectives of this research, which aims to fill this scientific gap.

In analyzing the two groups compared in this research, several points for discussion were highlighted, as described below:

As reported by Heinrichs and Zakzanis [20], schizophrenia affects several areas of brain processing, with varying degrees of impairment in many cognitive domains, including the ability to maintain attention, the speed of visual/auditory perception and processing, working memory, verbal and visual learning, abstract, spatial and numerical learning, pattern recognition, problem solving, rules of social coexistence and affectivity, among others, as described by Nuechterlein et al. [21]. In our investigation, most of these functionalities were tested with the aim of simulating real-life brain stress, with the main intention of evaluating behavior in relation to voltage levels and frequency rhythm, both in healthy brains and in brains with schizophrenia. The lack of similar studies with absolute values for voltage and frequency made it difficult to compare and discuss the results obtained, like Norman et al. [51].

All volunteers were encouraged to complete all tests. Still, the best times were for volunteers with a higher education, late-onset schizophrenia, and early treatment of the first symptoms, according to clinical data collected from each patient. Self-motivation remains an open question, as pointed out by Abram et al. [52]. About this, it seems that the time spent on mental effort reflects the volunteer’s remaining work capacity, that is, their cognitive provision. It was observed that volunteers with greater cognitive preservation took longer to perform mental effort. In contrast, volunteers with more significant cognitive deficits completed the activities more quickly, as their concern was to finish the task, perhaps due to anxiety or simply to “get rid” of the responsibility of the task to be performed. It appears that the avoidance of effort discussed by Gold et al. [53] was present in the group with schizophrenia. However, the question of avoidance remains open: is it aimed at protecting brain voltage levels, and if this effort limit is exceeded, would it cause an imbalance in the synaptic clefts (and consequently in voltage levels), causing chaos and, in turn, a dangerous imbalance for the brain of a patient with schizophrenia, potentially leading to a psychotic episode? The limits of each schizophrenic brain are unknown.

A brain with schizophrenia, stabilized in its electrolytes and in the ionic exchanges during its reasoning, does not change its schizophrenia condition, as stated by Ueno et al. [54]. However, what remains of these reasonings is what was preserved from their pre-schizophrenia cognitive capacity. Our findings indicate that more resilient and well-formed brains can maintain their cognitive integrity for longer periods, despite schizophrenia, in accordance with Onaolapo and Onaolapo [14], and the review of Rarinca et al. [55]. In addition to nutrition and other factors, it seems that schooling in the pre-schizophrenia phase is very important. It is intellectual capital, a ballast that they can gradually use up over the remaining time of their lives (if well cared for). It is as if the brain acquires a ballast, a stockpile of intelligence and memory, like intellectual capital that it can spend over the years that remain before the decline in the occurrence of schizophrenia, as pointed out by Ohi et al. [56]. On the other hand, for brains that are not well developed, the intellectual base or capital is small and quickly dwindles, and so it seems that anxiety, or simply the need to “get rid of things”, can be a symptom of impending decline.

Based on the analysis of the collected data, it is possible to infer that there is a relation between a good time in the Time Effort and the possibility of returning to secular life or professional life. Also, we believe that our findings shown in Figure 14 may help to investigate whether a professional can/may return to work (or not), reduce their working hours, or undergo vocational/professional rehabilitation, as suggested by Patmisari et al. [57]; or help to verify if the people with low sustained time effort should retire, as reported by Fond et al. [58] and Harvey et al.’s review [59]. It was observed that in the SCHIZ group, only 21% of them (only 7 individuals of the 33) achieved more than 80% of the effort time. Sometimes a part-time and better way may be considered, as commented by Marwaha and Johnson [60]. In 2015, Sahu [15] observed that around 90% of people with schizophrenia wanted to return to work or other activities, but this haste perceived in our study (anxiety or “get rid”) may reflect in practice whether the professional will still be able to maintain the quality of their work, or not, as commented by Averbeck et al. [61]. The challenges of returning to work depend on each person, as described by Martini et al. [62].

During the research, we observed that when brain electrolyte parameters are stabilized, the behavior also stabilizes (without outbursts of anger or depressive states), and the voltage levels tend to remain adequate for the chosen waveform (Alpha, 8–13 Hz) for longer, as described by Abenson [63]. On the other hand, volunteers with greater agitation did not exhibit the waveforms chosen for this study in the IR and FR phases. This reinforces the relationship that the more stable the electrolyte levels, the more stable the voltage levels and the more stable the mood, according to Torres et al. [64].

EEG measures the potential of large groups of neurons that are in synchrony. The higher the measured potential, the greater the synchrony. Considering this, in comparison to the HEALTHY group, the SCHIZ group presented the following:

Vt parameter level, 3.85% lower in the IR phase, and 7.89% lower in the FR phase;

Vr parameter level, 5.55% lower in the IR phase and 10.70% lower in the FR phase;

F parameter level, 14.91% lower in the IR phase and 16.35% lower in the FR phase.

This fact demonstrates that, even under medication and with stabilized behavior and emotions, a brain affected by schizophrenia does not exhibit the same potential as a healthy brain; and that, when subjected to mental exertion, its potential and frequency levels decrease even further. The dominant frequency (F) was the most affected parameter, according to Brandt et al. [65], Moran and Hong [66], and Vass et al. [67].

The hemispheric averages (median/mean) presented in the study aimed to assess the laterality of each group and compare voltage potentials between the groups, but the values were very similar. Points O1 and O2 were noteworthy for the SCHIZ group; in both the IR and FR phases, they exhibited a significantly lower potential than those of the HEALTHY group at both locations. Regarding the FR phase, the difference strongly corroborates the abnormalities cited in the literature that explain the loss of gray and white matter in the brain region, as commented by Tohid, Faizan, and Faizan [39].

The analysis of the frequency parameter showed that the SCHIZ group experienced disorganization when exposed to mental exertion, as reported by Brandt [65]. While the HEALTHY group also showed behavioral maladjustment, evidenced by the presence of several outliers, the SCHIZ group, which already had 15 outliers in IR, had 25 outliers in FR (an increase of 66.66%). This is a cause for concern (considering a return to work as already described by Abenson [63]), not only due to the observed disparity in the behavior of the frequency between the groups, but also in relation to the impact of mental exertion on a brain that already struggles at rest to maintain balance in its ionic exchanges. This explains and complements the significant difference observed in the time sustained during mental exertion, which varied considerably between the groups.

When the distribution is not normal and the sample is small, homoscedasticity is compromised, as it is not possible to make an inference about a single population. In this case, the median may indicate a trend. However, this research presents significant indicators about the population with schizophrenia with positive symptoms within the specifications of the research. We think that it is important to replicate the research to improve statistical significance and achieve better accuracy.

### Research Limitations

In this study, all volunteers with schizophrenia were on stable pharmacological regimens with one or more medications (sedatives, antidepressants, mood stabilizers, and antipsychotics, among others). It is known that these medications influence not only the correction of schizophrenia symptoms but also the levels of brain waves and brain wave patterns through their chemical interactions.

This study presented three main limiting factors:

(1) Long duration (approximately 3 h in total, including the time for EEG cable placement and the completion of the entire research routine);
(2) Reduced volunteer mobility (volunteers were asked to reduce movements to minimize artifacts and avoid poor contact with the electrodes, which were repeatedly tested);
(3) Low volunteer participation (due to the complexity of the research) impacted on the sample size. This is a factor that limits statistical accuracy.

Although the sample size was small due to the complexity of the research (movement restrictions to minimize artifacts and the duration of mental effort), the study demonstrates a significant advance toward the research objective. It should be replicated to improve statistical power and precision.

## 5. Conclusions

Despite being a demanding and lengthy study, no adverse events occurred with any volunteer during or after the experimental protocol.

From the results of the performed experimental essay, we concluded that in the Final Resting State (FR) phase, the EEG amplitude (Vtotal) was 7.89% lower for the schizophrenia group, and the EEG dominant frequency (F) was 16.35% lower than in the HEALTHY group.

Notable differences were observed in the tolerance parameter regarding the duration of sustained cognitive effort, in which 59% of the healthy volunteers reached the activities for the predefined time (75 min), and 100% of them had a performance greater than 90%. In the SCHIZ group, 6% reached the predefined time (75 min), and 21% had a performance greater than 80%.

As an extension to the conclusions, this investigation makes contributions to methodology and technology for mapping sustained mental effort for people with schizophrenia (or undefined, but a clinical suspicion must already exist), which can be used for evaluation of the evolution in treatment or for return to work or work-time adjustment.

Therefore, this study fills an important scientific gap, as it represents a conceptual shift in how individuals with schizophrenia can be assessed regarding their occupational functioning and how their work capacity can be quantified in terms of time.

## Figures and Tables

**Table 1:** Normality test for the Vt parameter and percentage of difference between mean and median by position of the EEG for HEALTHY (H) and SCHIZ (S) groups.

IR and FR *p*-Value of the Full Amplitude (Vt)					Difference Between Groups%			
Phase	IR	FR	IR	FR	IR	IR	FR	FR
Position	H *p* Value	H *p* Value	S *p* Value	S *p* Value	H_S Mean	H_S Median	H_S Mean	H_S Median
Fp1	0.82	0.91	0.26	<0.05	14.58	22.90	10.85	8.37
F3	0.07	0.62	0.21	0.79	32.68	15.59	8.77	4.78
F7	0.29	0.43	0.04	0.03	3.81	19.88	4.81	10.20
C3	0.95	0.08	0.33	0.59	20.91	30.88	1.57	7.04
T3	0.73	0.51	0.15	0.03	7.84	0.34	12.09	19.50
P3	0.68	0.79	0.53	0.79	3.46	3.20	9.82	17.01
T5	0.21	0.19	0.01	<0.05	7.90	12.81	11.07	15.73
O1	0.81	0.23	0.42	<0.05	16.13	27.67	23.99	39.24
A1	0.45	0.63	0.18	0.07	13.03	18.73	14.67	7.61
Fp2	0.84	0.35	<0.05	<0.05	3.76	9.46	15.42	15.61
F4	0.74	0.55	0.08	0.46	30.82	19.53	24.20	26.05
F8	0.48	0.85	0.12	0.13	0.57	1.03	3.80	5.36
C4	0.24	0.54	0.16	0.80	13.61	15.52	6.92	14.71
T4	0.06	0.48	0.01	0.10	6.46	18.07	13.83	15.20
P4	0.72	0.61	0.32	0.86	0.99	6.12	13.57	11.12
T6	0.30	0.55	0.67	0.26	8.05	16.11	17.92	16.59
O2	0.65	0.58	0.31	0.05	15.12	16.30	22.77	24.57
A2	0.22	0.02	0.51	0.87	26.29	29.09	16.84	29.79

Column with H refers to HEALTHY group data; Column with S refers to SCHIZ group data; Column H_S refers to the comparison between the groups.

**Table 2:** Normality test for the Vr parameter and percentage of difference between mean and median by position of the EEG for HEALTHY (H) and SCHIZ (S) groups.

IR and FR *p*-Value of the Effective Voltage (Vr)					Difference Between Groups%			
Phase	IR	FR	IR	FR	IR	IR	FR	FR
Position	H *p* Value	H *p* Value	S *p* Value	S *p* Value	H_S Mean	H_S Median	H_S Mean	H_S Median
Fp1	0.47	0.61	0.01	<0.05	16.96	22.83	6.74	8.41
F3	0.48	0.46	0.08	0.02	20.05	4.84	8.47	12.29
F7	0.41	0.60	0.11	0.17	3.51	9.77	11.52	15.12
C3	0.45	0.02	0.90	0.29	38.34	31.93	0.46	12.06
T3	0.61	0.08	0.13	0.12	7.82	0.00	18.08	17.20
P3	0.98	0.47	0.37	0.44	1.96	2.88	8.86	11.69
T5	0.54	0.74	<0.05	<0.05	9.39	13.03	14.69	17.45
O1	0.31	0.51	0.13	<0.05	21.07	31.37	26.95	46.70
A1	0.30	0.06	0.01	0.02	16.42	3.06	12.73	0.91
Fp2	0.68	0.09	<0.05	<0.05	2.57	6.53	9.39	6.90
F4	0.47	<0.05	0.01	0.15	25.40	24.87	30.68	16.33
F8	0.26	0.02	0.03	<0.05	3.47	1.50	7.72	17.19
C4	0.21	0.01	0.22	0.73	24.58	17.44	4.11	21.70
T4	0.23	0.34	0.02	0.07	5.21	12.17	17.42	21.04
P4	0.09	0.97	0.71	0.77	1.12	6.31	14.48	15.95
T6	0.61	0.87	0.61	0.11	10.62	18.52	23.61	24.67
O2	0.40	0.85	0.112	0.04	24.08	35.04	23.49	30.07
A2	0.17	<0.05	0.58	0.87	29.93	34.93	10.33	29.34

Column with H refers to HEALTHY group data; Column with S refers to SCHIZ group data; Column H_S refers to the comparison between the groups.

**Table 3:** Normality test for F parameter and percentage of difference between mean and median by position of the EEG for HEALTHY (H) and SCHIZ (S) groups.

IR and FR *p*-Value of the Dominant Frequency (F)					Difference Between Groups%			
Phase	IR	FR	IR	FR	IR	IR	FR	FR
Position	H *p* Value	H *p* Value	S *p* Value	S *p* Value	H_S Mean	H_S Median	H_S Mean	H_S Median
Fp1	0.77	<0.05	<0.05	0.11	14.10	14.71	24.94	24.24
F3	0.62	0.14	<0.05	0.05	11.77	16.28	20.57	21.21
F7	0.60	<0.05	<0.05	<0.05	14.44	15.15	21.09	24.24
C3	0.02	0.02	0.91	0.02	18.18	21.21	14.77	20.59
T3	0.09	0.02	<0.05	0.16	12.20	22.40	18.94	24.24
P3	<0.05	0.11	0.61	0.37	18.57	21.21	13.75	17.14
T5	0.20	0.16	0.31	0.37	11.91	11.76	8.83	11.11
O1	0.62	0.05	0.18	0.82	13.24	13.89	16.25	20.00
A1	<0.05	<0.05	<0.05	0.06	8.24	9.09	9.53	11.77
Fp2	0.63	<0.05	<0.05	0.01	14.67	25.81	22.95	24.24
F4	0.52	0.09	0.24	<0.05	21.60	25.00	21.03	24.24
F8	0.41	0.01	0.21	0.09	17.52	25.00	21.81	24.24
C4	0.18	0.11	0.03	0.04	13.99	14.29	23.05	28.13
T4	0.39	0.67	0.44	0.01	16.73	14.71	19.08	24.24
P4	0.52	0.01	<0.05	0.09	14.99	21.21	11.79	14.29
T6	0.04	0.05	0.25	0.02	17.08	22.40	11.53	17.65
O2	0.34	0.40	0.06	0.06	9.96	14.29	13.66	17.14
A2	<0.05	<0.05	<0.05	0.11	5.70	9.09	8.60	5.55

Column with H refers to HEALTHY group data; Column with S refers to SCHIZ group data; Column H_S refers to the comparison between the groups.

## Data Availability

The datasets presented in this article are not available because they are part of other ongoing studies and contain sensitive data that could compromise the privacy of the research volunteers.

## Abbreviations

The following abbreviations are used in this manuscript:

AG	Anti-Gliadin Antibodies
AgCl	Argentum Chloride (silver chloride)
ANOVA	Analysis of Variance
CT	Computed Tomography
EEG	Electroencephalogram
f	Frequency (Hz)
F	Dominant Frequency (Hz)
fMRI	Functional Magnetic Resonance Imaging
FR	Final Register
Ig	Immunoglobulin
IR	Initial Register
MRI	Magnetic Resonance Imaging
Vm	Voltage Mean (µV)
Vpp	Voltage Peak-to-Peak (µV)
Vr	Volt Root Mean Square (Vrms) (µV)
Vt	Voltage Total (µV)

## References

[1] Bressan R.A. Schizophrenia. Interview given to Varella, Drauzio. Video. São Paulo, SP, BR. 2012. Portuguese. https://www.youtube.com/watch?v=VsX7MnFY8JE.

[2] Voichcoski B.M., Neto V.M. Esquizofrenia: Uma doença Multifatorial. Proceedings of the 24º Congresso Brasileiro de Eng. Biomédica.

[3] Voichcoski B.M., Neto V.M. Schizophrenia: Interaction between factors. Proceedings of the World Conference on Medical Physics & Biomedical Engineering.

[4] Davis K.L., Stewart D.G., Friedman J.I., Buchsbaum M., Harvey P.D., Hof P.R., Buxbaum J., Haroutunian V. (2003). White matter changes in schizophrenia: Evidence for myelin-related dysfunction. Arch. Gen. Psychiatry.

[5] Susser E., St Clair D., He L. (2008). Latent effects of prenatal malnutrition on adult health: The example of schizophrenia. Ann. N. Y. Acad. Sci..

[6] Bell R.L., Hauser S.R., McClintick J., Rahman S., Edenberg H.J., Szumlinski K.K., McBride W.J. (2016). Ethanol-Associated Changes in Glutamate Reward Neurocircuitry: A Mini-review of Clinical and Preclinical Genetic Findings. Prog. Mol. Biol. Transl. Sci..

[7] Guest F.L., Martins-de-Souza D., Rahmoune H., Bahn S., Guest P.C. (2013). Os efeitos do estresse na função do eixo hipotalâmico-pituitário-adrenal em indivíduos com esquizofrenia. Arch. Clin. Psychiatry.

[8] Seth S., Lewis A.J., Galbally M. (2016). Perinatal maternal depression and cortisol function in pregnancy and the postpartum period: A systematic literature review. BMC Pregnancy Childbirth.

[9] Alvim P.H.P., Cavalari V.C., Bianchini A.J., Frizon G.F., Ferreira G.M., Savian H.F.H., Drehmer I., Goularte J.F., Garcia L.F.C., Carriello M. (2025). Toxoplasmosis infection and schizophrenia: Elevated IgM reactivity index as a predictor of symptom worsening in chronic schizophrenia. Schizophr. Res..

[10] Breitschwerdt E.B., Greenberg R., Maggi R.G., Mozayeni B.R., Lewis A., Bradley J.M. (2019). Bartonella henselae Bloodstream Infection in a Boy with Pediatric Acute-Onset Neuropsychiatric Syndrome. J. Cent. Nerv. Syst. Dis..

[11] DSM-IV Diagnostic and Statistical Manual of Mental Disorders. Fifth Edition. APA (American Psychiatric Association. https://www.physio-pedia.com/images/1/1b/Diagnostic_and_statistical_manual_of_mental_disorders_DSM-5_%28_PDFDrive.com_%29.pdf.

[12] Beck A.T. (2010). Terapia Cognitiva da esquizofrenia.

[13] Onaolapo O.J., Onaolapo A.Y. (2021). Nutrition, nutritional deficiencies, and schizophrenia: An association worthy of constant reassessment. World J. Clin. Cases.

[14] So M.T.-W., Ullah A., Waris A., Alhumaydhi F.A. (2025). The Etiological Role of Impaired Neurogenesis in Schizophrenia: Interactions with Inflammatory, Microbiome and Hormonal Signaling. Int. J. Mol. Sci..

[15] Sahu K.K. (2015). Intervening Negative Impact of Stigma on Employability of a Person with Schizophrenia Through Social Case Work. J. Psychosoc. Rehabil. Ment. Health.

[16] Toro C.T., Deakin J.F. (2007). Adult neurogenesis and schizophrenia: A window on abnormal early brain development?. Schizophr. Res..

[17] Costa E., Davis J., Grayson D.R., Guidotti A., Pappas G.D., Pesold C. (2001). Dendritic spine hypoplasticity and downregulation of reelin and GABAergic tone in schizophrenia vulnerability. Neurobiol. Dis..

[18] El Jabiry S.E., Bouazzaoui M.A., Barrimi M., Elghazouani F., Oneib B. (2025). Stress, coping strategies, and relapse among schizophrenia patients at the psychiatric hospital of Oujda, Morocco. Pan Afr. Med. J..

[19] Nakamura Y., Shimada K., Miyamoto R., Koreki A., Anamizu S., Mimura M. (2025). Impact of negative symptom reduction on employment and vocational activity outcomes following cognitive remediation therapy in schizophrenia: A 1-year follow-up study. PCN Rep..

[20] Heinrichs R.W., Zakzanis K.K. (1998). Neurocognitive deficit in schizophrenia: A quantitative review of the evidence. Neuropsychology.

[21] Nuechterlein K.H., Barch D.M., Gold J.M., Goldberg T.E., Green M.F., Heaton R.K. (2004). Identification of separable cognitive factors in schizophrenia. Schizophr. Res..

[22] Vetter T.R., Cubbin C. (2019). Psychometrics: Trust, but Verify. Anesth. Analg..

[23] Kumari A., Obazenu L.O., Monis M., Mustapha-Adebiyi H.T., Wandala A., Harischandra T.V., Ayuba P.D., Thorani M., Arshad M.R., Al Shamsi M. (2025). Association between iron deficiency without anemia and cognitive impairment in children. Cureus.

[24] Begić D., Popović-Knapić V., Grubišin J., Kosanović-Rajačić B., Filipčić I., Telarović I., Jakovljević M. (2011). Quantitative electroencephalography in schizophrenia and depression. Psychiatr. Danub..

[25] Fink M., Itil T., Clyde D. (1965). The classification of psychoses by quantitative EEG measures. Recent Adv. Biol. Psychiatry.

[26] Martino F., Amici G., Godi I., Baretta M., Biasi C., Carta M., Corradi V., De Cal M., Knust M., Tamayod C. (2021). Gadolinium-based contrast media exposure and the possible risk of subclinical kidney damage: A pilot study. Int. Urol. Nephrol..

[27] Mizutani R., Saiga R., Yamamoto Y., Uesugi M., Takeuchi A., Uesugi K., Terada Y., Suzuki Y., De Andrade V., De Carlo F. (2021). Structural diverseness of neurons between brain areas and between cases. Transl. Psychiatry.

[28] Dvey-Aharon Z., Fogelson N., Peled A., Intrator N. (2015). Schizophrenia detection and classification by advanced analysis of EEG recordings using a single electrode approach. PLoS ONE.

[29] Sponheim S.R., Clementz B.A., Iacono W.G., Beiser M. (1994). Resting EEG in first episode and chronic schizophrenia. Psychophysiology.

[30] Newson J.J., Thiagarajan T.C. (2019). EEG Frequency Bands in Psychiatric Disorders: A Review of Resting State Studies. Front. Hum. Neurosci..

[31] Hunt M.J., Kopell N.J., Traub R.D., Whittington M.A. (2017). Aberrant Network Activity in Schizophrenia. Trends Neurosci..

[32] Galderisi S., Mucci A., Volpe U., Boutros N. (2009). Evidence-based medicine and electrophysiology in schizophrenia. Clin. EEG Neurosci..

[33] Howells F.M., Temmingh H.S., Hsieh J.H., van Dijen A.V., Baldwin D.S., Stein D.J. (2018). Electroencephalographic delta/alpha frequency activity differentiates psychotic disorders: A study of schizophrenia, bipolar disorder and methamphetamine-induced psychotic disorder. Transl. Psychiatry.

[34] Wang X., Chen S., Li J., Gao Y., Li S., Li M., Liu X., Liu S., Ming D. (2025). Enhanced theta oscillations in the left temporoparietal region associated with refractory positive symptoms in schizophrenia. Schizophrenia.

[35] Ramsay I.S., Lynn P.A., Schermitzler B., Sponheim S.R. (2021). Individual alpha peak frequency is slower in schizophrenia and related to deficits in visual perception and cognition. Sci. Rep..

[36] Renaldi R., Kim M., Lee T.H., Kwak Y.B., Tanra A.J., Kwon J.S. (2019). Predicting Symptomatic and Functional Improvements over 1 Year in Patients with First-Episode Psychosis Using Resting-State Electroencephalography. Psychiatry Investig..

[37] Elvevåg B., Wynn R., Covington M.A. (2011). Meaningful confusions and confusing meanings in communication in schizophrenia. Psychiatry Res..

[38] Jang K.I., Lee C., Lee S., Huh S., Chae J.-H., Huh S., Chae J.-H. (2020). Comparison of frontal alpha asymmetry among schizophrenia patients, major depressive disorder patients, and healthy controls. BMC Psychiatry.

[39] Tohid H., Faizan M., Faizan U. (2015). Alterations of the occipital lobe in schizophrenia. Neurosciences.

[40] Shao Y.R., Kahali P., Houle T.T., Deng H., Colvin C., Dickerson B.C., Brown E.N., Purdon P.L. (2020). Low Frontal Alpha Power Is Associated with the Propensity for Burst Suppression: An Electroencephalogram Phenotype for a “Vulnerable Brain”. Anesth. Analg..

[41] Mierau A., Klimesch W., Lefebvre J. (2017). State-dependent alpha peak frequency shifts: Experimental evidence, potential mechanisms and functional implications. Neuroscience.

[42] Klimesch W. (1999). EEG alpha and theta oscillations reflect cognitive and memory performance: A review and analysis. Brain Res. Brain Res. Rev..

[43] de Sena E.P., Sampaio A.S., Quarantini L.d.C., de Oliveira I.R. (2003). Diabetes mellitus e antipsicóticos atípicos. Braz. J. Psychiatry.

[44] Aleman M., Costa L.R.R., Crowe C., Kass P.H. (2018). Presumed Neuroglycopenia Caused by Severe Hypoglycemia in Horses. J. Vet. Intern Med..

[45] Das Chakladar D., Roy P.P. (2024). Cognitive workload estimation using physiological measures: A review. Cogn. Neurodyn..

[46] Sortwell A., Gkintoni E., Díaz-García J., Ellerton P., Ferraz R., Hine G. (2026). Beyond Cognitive Load Theory: Why Learning Needs More than Memory Management. Brain Sci..

[47] Finan D.A., McCann T.W., Rhein K., Dassau E., Breton M.D., Patek S.D., Anhalt H., Kovatchev B.P., Doyle F.J., Anderson S.M. (2014). Effect of Algorithm Aggressiveness on the Performance of the Hypoglycemia-Hyperglycemia Minimizer (HHM) System. J. Diabetes Sci. Technol..

[48] Jurgens H.A., Johnson R.W. (2012). Dysregulated neuronal-microglial crosstalk during aging, stress, and inflammation. Exp. Neurol..

[49] Cheng K.-Y., Robinson N., Ploner A., Kuja-Halkola R., Molero Y., Lichtenstein P., Bergen S.E. (2024). Impact of traumatic brain injury on risk for schizophrenia and bipolar disorder. Psychiatry Res..

[50] Černis E., Vassos E., Brébion G., McKenna P.J., Murray R.M., David A.S., MacCabe J. (2015). Schizophrenia patients with high intelligence: A clinically distinct subtype of schizophrenia?. Eur. Psychiatry.

[51] Norman R.M., Malla A.K., Williamson P.C., Morrison-Stewart S.L., Helmes E., Cortese L. (1997). EEG coherence and syndromes in schizophrenia. Br. J. Psychiatry.

[52] Abram S.V., Weittenhiller L.P., Bertrand C.E., McQuaid J.R., Mathalon D.H., Ford J.M., Fryer S.L. (2022). Psychological Dimensions Relevant to Motivation and Pleasure in Schizophrenia. Front. Behav. Neurosci..

[53] Gold J.M., Kool W., Botvinick M.M., Hubzin L., August S., Waltz J.A. (2015). Cognitive effort avoidance and detection in people with schizophrenia. Cogn. Affect Behav. Neurosci..

[54] Ueno Y., Aoki N., Yabuki T., Kuraishi F. (1961). Electrolytes metabolism in blood and cerebrospinal fluid in psychoses. Psychiatry Clin. Neurosci..

[55] Rarinca V., Vasile A., Visternicu M., Burlui V., Halitchi G., Ciobica A., Singeap A.-M., Dobrin R., Burlui E., Maftei L. (2024). Relevance of diet in schizophrenia: A review focusing on prenatal nutritional deficiency, obesity, oxidative stress, and inflammation. Front. Nutr..

[56] Ohi K., Sumiyoshi C., Fujino H., Yasuda Y., Yamamori H., Fujimoto M., Sumiyoshi T., Hashimoto R. (2017). A Brief Assessment of Intelligence Decline in Schizophrenia as Represented by the Difference between Current and Premorbid Intellectual Quotient. Front. Psychiatry.

[57] Patmisari E., Huang Y., Orr M., Govindasamy S., Hielscher E., McLaren H. (2024). Supported employment interventions with people who have severe mental illness: Systematic mixed-methods umbrella review. PLoS ONE.

[58] Fond G., Dubreucq J., de Verville P.L.S., Godin O., Andrieu-Haller C., Berna F., Aouizerate B., Capdevielle D., Chereau I., Clauss-Kobayashi J. (2022). Early-life factors associated with increased risk of disability pension in the national real-world schizophrenia FACE-SZ cohort study. Eur. Arch. Psych. Clin. Neurosci..

[59] Harvey P.D., Bosia M., Cavallaro R., Howes O.D., Kahn R.S., Leucht S., Müller D.R., Penadés R., Vita A. (2022). Cognitive dysfunction in schizophrenia: An expert group paper on the current state of the art. Schizophr. Res. Cogn..

[60] Marwaha S., Johnson S. (2004). Schizophrenia and employment—A review. Soc. Psychiatry Psychiatr. Epidemiol..

[61] Averbeck B.B., Evans S., Chouhan V., Bristow E., Shergill S.S. (2011). Probabilistic learning and inference in schizophrenia. Schizophr. Res..

[62] Martini L.C., Lussi I.A.d.O., Magalhães L., Santos F.V., Pimentel F.d.A., Petreche M.B., Fonseca A.O., Attux C., Bressan R.A. (2019). Experiência laboral e inclusão social de indivíduos com esquizofrenia. Rev. Bras. Saúde Ocup..

[63] Abenson M.H. (1970). EEGs in Chronic Schizophrenia. Br. J. Psychiatry.

[64] Torres S.J., Nowson C.A., Worsley A. (2008). Dietary electrolytes are related to mood. Br. J. Nutr..

[65] Brandt C.L., Kaufmann T., Agartz I., Hugdahl K., Jensen J., Ueland T., Haatveit B., Skatun K.C., Doan N.T., Melle I. (2015). Cognitive Effort and Schizophrenia Modulate Large-Scale Functional Brain Connectivity. Schizophr. Bull..

[66] Moran L.V., Hong L.E. (2011). High vs. low frequency neural oscillations in schizophrenia. Schizophr. Bull..

[67] Vass Á., Pálffy Z., Sörnyei D., Lányi O., Pongrácz M., Szabó G., Császár I., Csukly G., Farkas K. (2026). EEG spectral power across rest, body scan, and task conditions in Autism and Schizophrenia. Psychiatry Res. Neuroimaging.

